# The Role of TRP Channels in Colitis and Inflammatory Bowel Disease: A Systematic Review

**DOI:** 10.3390/ijms26199390

**Published:** 2025-09-25

**Authors:** Kristina A. Dvornikova, Olga N. Platonova, Elena Y. Bystrova

**Affiliations:** I.P. Pavlov Institute of Physiology RAS, 199034 St. Petersburg, Russia; 691442@gmail.com (K.A.D.);

**Keywords:** TRP channels, inflammatory bowel disease, ulcerative colitis, Crohn’s disease, colitis, inflammation

## Abstract

Comprising ulcerative colitis (UC) and Crohn’s disease (CD), inflammatory bowel disease (IBD) denotes a series of long-standing, relapsing inflammatory disorders of the digestive tract. There is increasing evidence in the literature indicating that IBD pathogenesis is associated with the dysfunction of ion channels, with Transient Receptor Potential (TRP) channels being of particular importance. Through this systematic review, the significance of various TRP channel types in the pathogenesis of colitis and IBD will be appraised. A comprehensive literature search was conducted in PubMed, ScienceDirect, and Google Scholar, encompassing original research articles, using the principles of the PRISMA statement (last search: 15 May 2025). The search terms used were “Transient Receptor Potential Channels”, “TRP channels”, “TRPV1”, “TRPA1”, “TRPV4”, “TRPV2”, “TRPM2”, “TRPM3”, “TRPM7”, “TRPM8”, “TRPC3”, “colitis”, “inflammatory bowel disease”, “IBD”, “ulcerative colitis”, “Crohn Disease”. A total of 48 studies met the inclusion criteria. Risk of bias was assessed using SYRCLE’s Risk of Bias tool for preclinical studies and the GRADE approach for clinical studies. According to a review of the literature, some TRP channels may exhibit contradictory effects when evaluating pain sensitivity or inflammation, while no conflicting effects have been observed for other TRP channels. Thus, TRPV1 and TRPA1 channels demonstrated opposing effects on pain sensitivity, but TRPV4, TRPM2, TRPM3, and TRPM8 were exclusively linked to elevated pain. Only anti-inflammatory activity was shown for TRPV3, TRPC1, and TRPC6 channels. In contrast, TRPV6, TRPM2, and TRPM3 channels were exclusively associated with a pro-inflammatory role. Concurrently, both pro- and anti-inflammatory effects were manifested for TRPA1, TRPV1, TRPV4, and TRPV5. The literature suggests that these TRP channels exert significant and diverse effects on the pathophysiology of colitis and IBD. Understanding the specific contributions of each TRP channel may pave the way for the development of targeted therapeutic interventions aimed at controlling inflammation and alleviating the symptoms of IBD. This systematic review was funded by the Russian Science Foundation (grant #24-25-00267).

## 1. Introduction

Inflammatory bowel disease (IBD), including Crohn’s disease (CD) and ulcerative colitis (UC), is a spectrum of chronic and recurrent inflammatory diseases that affect the gastrointestinal tract (GI) and is a significant global health problem affecting millions of people worldwide [1,2,3]. IBD pathogenesis is multi-faceted, including genetic predisposition, environmental triggers, mucosal immune dysregulation and gut microbial changes [4]. UC primarily affects the colon with superficial inflammation, leading to diarrhea and blood in stool, with periods of exacerbation and remission. CD can affect any part of the GI tract with transmural inflammation, causing pain, diarrhea, obstruction, and perianal lesions. Both involve complex and dysregulated immune responses and microbiome alterations. Therapy ranges from 5-aminosalicylic acid (5-ASA) and corticosteroids for mild UC to immunosuppressants and biologics targeting TNF-α, IL-12, and IL-23 for severe disease and CD [1,2,3,4].

Transient receptor potential (TRP) channels, a superfamily of non-selective cationic channels, are increasingly being studied in the pathophysiology of colitis and IBD [5,6], and some of these channels are now recognized as promising targets for developing treatment strategies in IBD. Polycystin (TRPP or PKD), canonical (TRPC), melastatin (TRPM), vanilloid (TRPV), ankyrin (TRPA), and mucolipin (TRPML) are the six subfamilies that belong to this ion channel group, which consists of 28 members. These tetrameric channels are made up of six transmembrane-spanning proteins that exhibit a similar structure. Differences in the cytosolic N- and C-terminals are what distinguish family members from one another [5,6,7,8,9,10,11,12,13,14,15,16,17]. TRP channels are widely distributed throughout the GI and perform various functions, including supporting visceral and somatic nociception and maintaining the physiological function of the GI [18,19]. Neurogenic inflammation, or inflammation induced by local release of immunomodulatory neuropeptides, may be induced by activation of the TRP channels [20,21]. Many immune cells also express certain TRP channels that are primarily responsible for modulatory processes such as phagocytic activity, cell migration, and cytokine release [19].

In recent years, studies have emerged examining the role of some of these channels in the pathogenesis of IBD, namely TRPA1 [22,23,24,25,26,27,28,29,30,31,32,33,34,35,36], TRPV1-6 [5,24,25,26,37,38,39,40,41,42,43,44,45,46,47,48,49,50,51,52,53,54,55,56,57,58], TRPM2, 3, and 8 [5,59,60,61,62,63,64,65,66,67], and TRPC1 and 6 [5,68]. TRPA1 is a non-selective cation channel that is the only member of the ankyrin subfamily. It is predominantly expressed in sensory neurons, where it acts as a key molecular sensor of noxious stimuli, including endogenous pro-inflammatory mediators [69].

Based on functional traits, the six members of the TRPV subfamily can be further divided into two fairly different groups. Involved in sensory processes like thermosensation, chemosensation, and nociception, TRPV1–4 are nonselective cation channels that are gated by chemical or physical stimuli. Conversely, TRPV5 and TRPV6 are calcium-selective epithelial channels that help the kidney and intestines absorb calcium, which is crucial for preserving calcium homeostasis [70,71]. Additionally, TRPV5 and TRPV6 are highly expressed in the GI and were also detected in the pancreas, testis, prostate, placenta, brain, and salivary gland [72].

The eight members of the TRPM subfamily, which range from TRPM1 to TRPM8, are involved in many physiological and pathological aspects, especially in cell development and ion homeostasis. To regulate these processes, TRPM channels stimulate intracellular Ca^2+^ signaling in response to lipid, ion concentration, or second messengers. TRPM channels can be activated by a variety of physical or chemical stimuli, including temperature, osmolality, and pH [12,13,73,74]. There is evidence that TRPM2 is expressed in mucosal macrophages and mast cells in the GI and is involved in the progression of experimental colitis and food allergy [59]. TRPM3 has been shown to be expressed in a variety of tissues including the nervous system, adipocytes, pancreatic β-cells, kidney, retina, and pituitary gland, where it is involved in thermosensory transmission and modulation of nociceptive signaling [12,13,75]. TRPM8 is involved in the gastrointestinal physiology and pathophysiology [76]. Notably, TRPM8 activation exerts a protective effect against indomethacin-induced small intestinal injury via the release of calcitonin gene-related peptide (CGRP), highlighting its relevance in intestinal mucosal defense mechanisms [15].

TRPC subfamily is a group of receptor-operated calcium-permeable nonselective cation channels which includes seven receptor-activated channels, but in humans the *trpc2* gene has become a pseudogene, resulting in only six TRPC proteins being expressed [77,78]. These TRPCs can be activated by G protein-coupled receptors (GPRs), protein kinases, or mechanical stimuli, subsequently leading to cell depolarization [78,79,80]. Additionally, TRPC6 channels are reported to permeate metal ions such as Zn^2+^ and Fe^2+^ [68]. Thus, the effects of different subtypes of TRP channels appear to be different [6], and further study of their role in the context of IBD pathogenesis may contribute to the development of new therapeutic approaches in the treatment of this pathology.

The goal of this systematic review is to thoroughly assess the available data about the function of these TRP channels in colitis and IBD, with an emphasis on how they relate to inflammatory signaling pathways and pain perception. We aim to clarify the roles of TRP channels in the development and manifestation of IBD by combining data from colitis animal models and patients with UC or CD.

## 2. Materials and Methods

### 2.1. Review Framework and Objectives

This systematic review was carried out in accordance with PRISMA guidelines [81] and structured according to the PICO (Population, Intervention, Comparison, Outcome) model to address the central research objective:

In animal models or patients with colitis or IBD, do TRP channels contribute to the development, progression, or modulation of inflammation and pain compared to conditions without TRP channel involvement?

P (Population): Animals or patients with colitis or IBD, including Crohn’s disease and ulcerative colitis.

I (Intervention/Exposure): Activation, inhibition, or expression analysis of TRP channels.

C (Comparison): Absence of TRP channel modulation, knockout models, or untreated controls.

O (Outcome): Changes in intestinal inflammation, pain sensitivity, histological damage, cytokine levels, or clinical severity.

The detailed PRISMA Main Checklist is available in Supplementary Table S1.

### 2.2. Literature Search Strategy

A comprehensive literature search was performed using three major databases: PubMed, ScienceDirect, and Google Scholar. The following Boolean query was applied across all platforms:

((“Transient Receptor Potential Channels”[Title/Abstract] OR “TRP channels”[Title/Abstract] OR TRPV1[Title/Abstract] OR TRPA1[Title/Abstract] OR TRPV4[Title/Abstract] OR TRPV2[Title/Abstract] OR TRPM2[Title/Abstract] OR TRPM3[Title/Abstract] OR TRPM7[Title/Abstract] OR TRPM8[Title/Abstract] OR TRPC3[Title/Abstract]) AND (colitis[Title/Abstract] OR “inflammatory bowel disease”[Title/Abstract] OR IBD[Title/Abstract] OR “ulcerative colitis”[Title/Abstract] OR “Crohn Disease”[Title/Abstract])) AND (english[Language]) NOT (review[Publication Type])

All searches were carried out on 15 May 2025.

### 2.3. Study Selection Process

**Figure 1:**
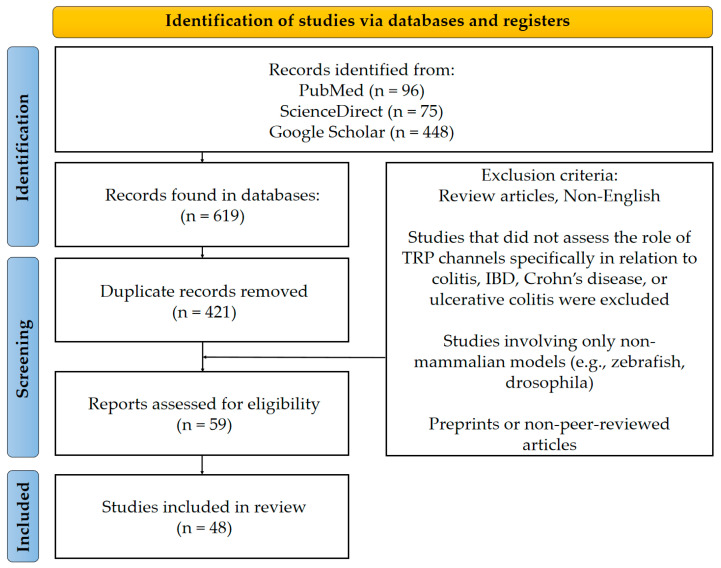
PRISMA flowchart with article selection process.

The study selection process is illustrated in Figure 1 using a PRISMA flowchart.

The selection included articles which could potentially fulfill the inclusion criteria: original research articles; studies of TRP channels; relevance for IBD, including CD, UC or colitis experimental models; studies of any kind, including in vitro human or animal cell studies, ex vivo tissue analysis or animal studies. Only peer reviewed English-language articles were considered. No restrictions were imposed as to the date of article of the included studies. For non-open access articles, the full text was obtained via institutional subscription services. The exclusion criteria included: review articles; non-English articles; studies not specifically addressing the role of TRP channels in colitis, IBD, CD or UC; studies using exclusively non-mammalian models (e.g., zebrafish, drosophila); preprints or sources not peer reviewed.

Titles and abstracts were screened independently by two reviewers (K.A.D. and E.Y.B.) to identify studies that met inclusion criteria. In case of disagreement between two reviewers, a third reviewer (O.N.P) was involved. All disputes have also been settled through negotiation.

Following the screening process, 48 original research articles met all inclusion criteria and were selected for qualitative synthesis. Among these, 40 research examined a single TRP channel, while 8 studies investigated several TRP channels (e.g., TRPV1 and TRPA1). Notably, 4 of the 48 articles included both animal models and human samples, and assessed different TRP channels, thereby contributing data to multiple Subsections (Section 3.1 and Section 3.2). To ensure methodological transparency, these studies have been counted separately under each of the relevant headings, leaving the total number of unique articles at 48.

In this systematic review, «single-channel studies» denote articles examining only one TRP channel, while «multi-channel studies» indicate studies analyzing two or more TRP channels. This terminology is unrelated to electrophysiological single-channel recordings. In addition, some studies combined human samples and animal models; these were designated as human + animal studies to reflect the inclusion of data from both species. For multi-channel studies, each TRP channel dataset was extracted and analyzed separately in the corresponding subsection, while the original article was counted only once in the total number of articles covered. Taking into account all such duplicates at the dataset level and cross-references, the total number of individual dataset items included in the quality summary was 66.

Where a single article presented results for more than one type of TRP channel or model, each distinct dataset was considered to be an independent analysis unit according to the Cochrane methodological guidance [82]. This strategy allowed for a more granular assessment of studies findings and better accommodated the heterogeneity in experimental designs, models, and outcomes. Consequently, the total number of items in the dataset exceeds the number of unique articles.

## 3. Results

### 3.1. TRP Channels and IBD in Humans

The analysis included eleven original research articles investigating the function of the TRP channel in healthy subjects and in IBD patients [5,22,23,37,38,39,40,42,43,44]. Since four of these studies included results from animal models as well as human samples, these are also discussed in Section 3.2 [22,37,38,39]. Four articles were multi-channel studies, and seven were single-channel studies. In addition, four studies examining different channels were duplicated in Section 3.1 ([22] (duplicated once); [40] (duplicated twice); [5] (duplicated three times); [41] (duplicated four times)). A total of 21 dataset entries were analyzed in this Subsection (Table 1).

The level of evidence of the studies was assessed according to the GRADE system [83]. The GRADE system classifies evidence into four quality levels: high, moderate, low, and very low. High-quality evidence suggests that it is unlikely that further research will change the confidence in the estimated effect. Moderate-quality evidence suggests that further research is likely to have an important impact on confidence of the estimate and may change it. Low-quality evidence means that further research is very likely to have an important impact on confidence in the estimation and may alter it. Very low-quality evidence makes any estimate of the effect highly uncertain. The evaluation found that of the eleven studies reviewed, seven were rated as having a “very low” level of evidence, while four were rated as “low” (Supplementary Table S2). All eleven original research articles included in the analysis are observational.

**Figure 2:**
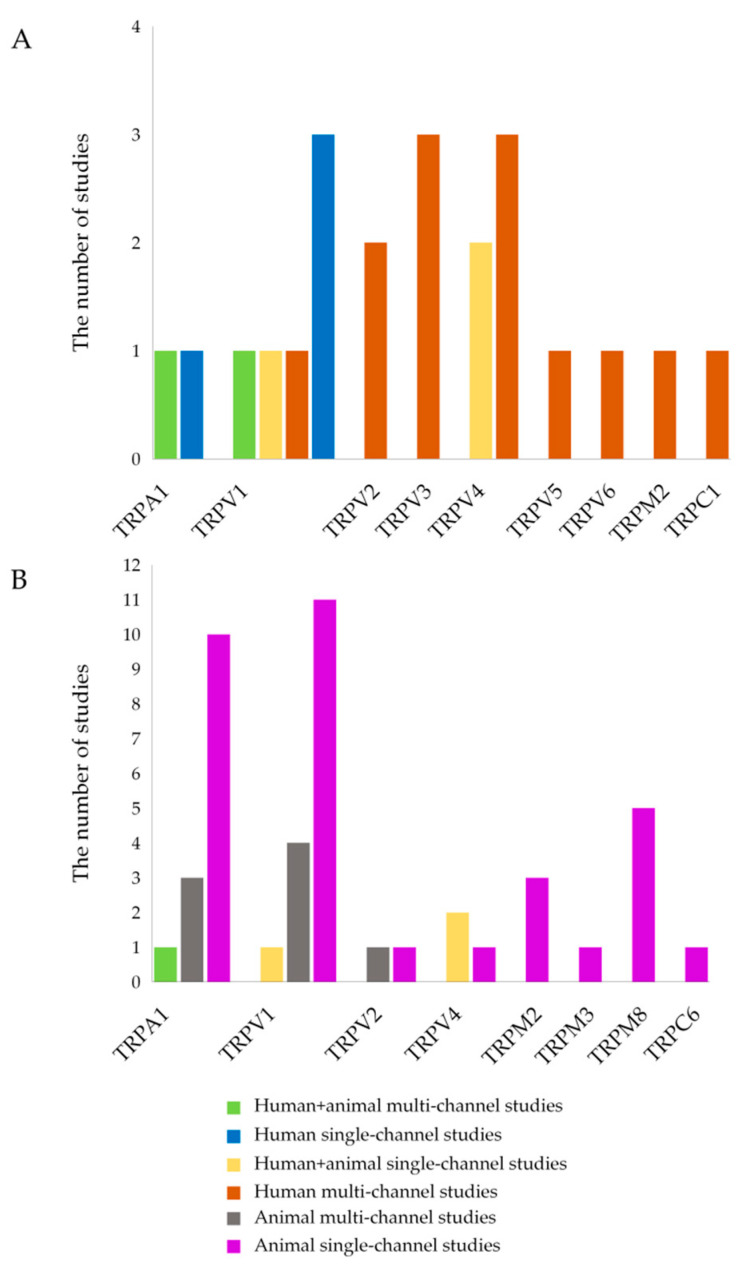
The categorization and distribution of TRP-related studies addressed in Section 3.1 and Section 3.2. (**A**) Human study types. (**B**) Animal study types.

The categorization and distribution of human studies are presented in Figure 2A, where each study type (e.g., human + animal multi-channel studies, human single-channel studies, etc.) is visualized by a specific color.

#### 3.1.1. Effects of TRPA1 in Humans

In the study by Kun et al. [22] examining colon biopsies from UC and CD patients, TRPA1 expression was significantly higher in patients with active IBD than in patients with inactive IBD, suggesting that TRPA1 function is switched to reduce inflammation in the acute phase and that it may play a protective role due to downregulation of substance P (SP), neurokinin A (NKA), neurokinin B (NKB), tumor necrosis factor alpha (TNF-α) genes that are involved in the development of IBD. Increased methylation of the TRPA1 promoter has been shown in another study examining whole blood samples, which was associated with dysregulated TRPA1 expression and elevated sensitivity to peripheral pain in CD patients. It was also shown that women perceive pain more sensitively than males do, indicating a gender-specific effect [23].

#### 3.1.2. Effects of TRPV1 in Humans

Most studies utilizing colon biopsies from humans with active and/or remission CD and UC indicate a pro-inflammatory role for TRPV1 in IBD patients and point to an association of this channel with increased inflammation, abdominal pain and visceral hypersensitivity (VHS) [37,42,43]. Specifically, quiescent IBD patients with abdominal discomfort and irritable bowel syndrome-like (IBS)-like symptoms had more TRPV1-positive nerve fibers than asymptomatic quiescent CD and UC patients, and this number was positively correlated with the intensity of the pain [43]. TRPV1 gene expression was found to be higher in UC patients in remission than in those with active UC. This result suggests a negative correlation between TRPV1 gene expression and the degree of inflammation. Additionally, it was shown that a higher level of TRPV1 gene expression correlated to both an earlier diagnosis and a recurrent illness course. All intestinal layers of active UC patients concurrently showed higher levels of TRPV1 protein than non-IBD controls, confirming that the protein is involved in the inflammatory response. The inconsistency between gene expression and protein level in the context of particular pathogenic scenario can be explained by a variety of factors, such as complicated gene regulatory systems, RNA transport, post-transcriptional modifications, and mRNA degradation [42]. Similarly, another study demonstrated that TRPV1 mRNA is significantly reduced in the colon tissue of patients with active IBD compared to the non-inflamed samples [22]. Luo et al. reported more intense TRPV1 immunoreactivity in the colonic epithelium of patients with active IBD; however, TRPV1 expression did not significantly correlate with disease duration or activity [44]. It has been reported that patients with active UC had statistically lower levels of TRPV1 expression compared to the control group. A significant decrease in TRPV1 expression levels may be associated with the exacerbated colon inflammation. In addition, there was no convincing evidence on its correlation with clinical outcomes and disease severity. Furthermore, a significant positive correlation was identified between TRPV1 and TRPV3 expression levels in UC samples, indicating their potential synergistic effects in IBD [40].

#### 3.1.3. Effects of TRPV2 in Humans

Both UC and CD patients’ PBMCs demonstrated lower TRPV2 mRNA expression levels, which negatively correlated with disease activity in both groups. This suggests that TRPV2 may play a part in modulating inflammation. It is hypothesized that this reduction could lessen the pro-inflammatory response and, consequently, the intensity of intestinal inflammation [5]. Similarly, Toledo Mauriño et al. found that colon tissue samples from patients with UC in both the active and remission occasions had lower TRPV2 gene expression than controls. Furthermore, when comparing controls to UC patients, TRPV2 protein levels were lower in the muscularis and serosa but higher in the mucosa and submucosa [41].

#### 3.1.4. Effects of TRPV3 in Humans

Morita et al. demonstrated that TRPV3 mRNA expression levels were lower in CD patients’ PBMCs than in healthy controls [5]. Additionally, there was a negative correlation between the UC group’s leukocyte count and TRPV3 mRNA expression levels [5]. In addition, TRPV3 gene expression and protein levels were higher in colon biopsies of controls than in patients with active UC, suggesting that disease activity is associated with downregulated TRPV3 expression [41]. Another study reported no significant difference in TRPV3 expression levels between UC and control colonic samples; however, a positive correlation was identified between TRPV1 and TRPV3 expression levels and TRPV3 and TRPV4 expression levels in UC patients, suggesting potential synergy [40].

#### 3.1.5. Effects of TRPV4 in Humans

The reported outcomes include heightened TRPV4 mRNA expression levels in PBMCs of CD patients compared with healthy controls, positive correlation of TRPV4 mRNA expression with the serum albumin level in the UC group and with the C-reactive protein (CRP) level in the CD group, suggesting a potential role for leukocyte TRPV4 in the pathophysiology of IBD [5]. Fichna et al. demonstrated that TRPV4 mRNA expression was significantly elevated in colon biopsies from patients with CD and UC compared with healthy subjects (2.9 and 4.5-fold, respectively), thus suggesting its crucial role in the intestinal inflammation [38]. Furthermore, TRPV4 expression in colonic epithelium of UC patients was higher compared to non-IBD controls, and a positive correlation was found between TRPV3 and TRPV4 expression levels in UC samples. However, no significant correlation was found between TRPV4 expression and disease severity. Nevertheless, the role of TRPV4 in the inflammatory process may be substantial [40]. Furthermore, the pro-inflammatory role of TRPV4 is discussed by D’Aldebert et al. [39]. In particular, elevated levels of TRPV4 were detected in Caco-2 cells and in epithelial cells of human colon tissue samples, where administration of the TRPV4 agonist 4α-phorbol-12,13-didecanoate (4αPDD) caused a dose-dependent increase in intracellular Ca^2+^ concentration and chemokine release. It is hypothesized that receptor activation may affect the innate immunity by maintaining the pro-inflammatory phenotype of intestinal epithelial cells and sustaining chronic inflammation. In contrast, Toledo Mauriño et al. demonstrated increased TRPV4 gene expression in remission UC patients compared to active UC subjects, as well as increased TRPV4 protein expression in all intestinal layers in the control group compared to patients with UC. Thus, these results indicate a positive correlation between TRPV4 expression and the health of the colon [41].

#### 3.1.6. Effects of TRPV5 in Humans

The study by Toledo Mauriño et al. [41] has shown that colonic tissue samples from active UC patients have lower levels of TRPV5 mRNA than healthy controls. The UC group also showed decreased TRPV5 protein levels in all intestinal layers. These results imply that TRPV5 deficiency may be associated with UC induction.

#### 3.1.7. Effects of TRPV6 in Humans

TRPV6 gene expression was significantly higher in colonic tissue samples of active UC patients compared to healthy controls. Furthermore, TRPV6 protein expression was increased in all intestinal layers of the colonic tissue biopsies from the UC patients including mucosa, submucosa, muscular layer, and serosa, thus suggesting TRPV6 involvement in the disease activity [41].

#### 3.1.8. Effects of TRPM2 in Humans

The study by Morita et al. [5] utilized samples comprising PBMCs from patients with UC and CD. The reported outcomes include heightened TRPM2 mRNA expression levels in PBMCs of UC and CD patients compared to healthy controls, indicating its possible pro-inflammatory role.

#### 3.1.9. Effects of TRPC1 in Humans

The same study [5] has shown that TRPC1 mRNA expression levels were lower in PBMCs of UC and CD patients compared with controls, suggesting that decreased TRPC1 expression may contribute to disease progression.

### 3.2. TRP Channels and Colitis in Animal Models

The analysis included 41 original research articles [22,24,25,26,27,28,29,30,31,32,33,34,35,36,37,38,39,45,46,47,48,49,50,51,52,53,54,55,56,57,58,59,60,61,62,63,64,65,66,67,68] investigating the role of TRP channels in experimental colitis models, including 4 articles that examined both humans and animals and therefore also discussed in Section 3.1 [22,37,38,39]. Five articles are multi-channel studies, whereas 36 articles are single-channel studies. Furthermore, because four multi-channel studies focus on different channels, they are duplicated in Section 3.2 [24,25,26,45]. A total of 45 records of the dataset were analyzed in this Subsection (Table 2).

**Figure 3:**
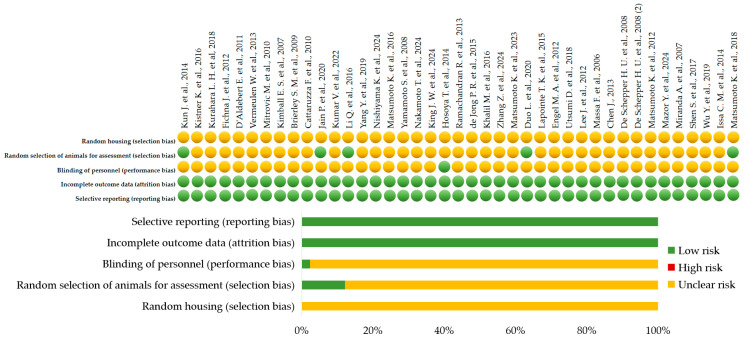
A risk of bias (SYRCLE’s RoB criteria) summary and diagram including animal studies [22,24,25,26,27,28,29,30,31,32,33,34,35,36,37,38,39,45,46,47,48,49,50,51,52,53,54,55,56,57,58,59,60,61,62,63,64,65,66,67,68].

For preclinical studies, the SYRCLE’s Risk of Bias tool was used [84]. In this review, the risk of bias for preclinical studies was assessed using SYRCLE’s RoB tool [84] across five domains (random housing, random selection of animals for assessment, blinding of personnel, incomplete outcome data, selective reporting) (Figure 3). All graphical visualizations were generated using Microsoft Excel (Office 2021 Pro Plus).

Low risk of bias was assigned when the methodological approach for a given domain was explicitly described and met the SYRCLE’s RoB criteria. High risk of bias was assigned when the study clearly indicated that the methodological safeguard was not implemented. Unclear risk of bias was assigned when there was insufficient information to determine whether the safeguard was implemented. The absence of reporting was not considered evidence that the measure was not performed; therefore, if randomization, blinding, or other safeguards were not mentioned and not explicitly excluded, the risk was classified as unclear. Among the 41 original research articles, only six studies (14.6%) were judged to have a low risk of bias across all evaluated domains (Figure 3) (Supplementary Table S3) [22,29,33,37,58,63]. The remaining 35 studies (85.4%) were deemed to have an unclear risk of bias, mainly because they lacked blinding and randomization.

In animal studies, specific pathogen free (SPF) status was additionally assessed by four criteria: verified SPF certificate, controlled environment, genetic homogeneity, complete documentation [85,86]. Studies with unrestricted animal care or no confirmed SPF status were deemed to have a high risk of bias (red circle); the ones with confirmed SPF status but insufficient information about animal care were evaluated as having a moderate risk of bias (yellow circle); and those with confirmed SPF status and comprehensive information about animal care were considered to have a low risk of bias (green circle). 25 articles (61.0%) of the 41 preclinical studies were categorized as moderate risk (yellow circle), and 16 articles (39%) as high risk (red circle). There were no studies that met all four requirements for being classified as low risk (green circle) (Supplementary Table S3).

The categorization and distribution of animal studies are presented in Figure 2B, where each study type (e.g., human + animal multi-channel studies, animal single-channel studies, etc.) is visualized by a specific color.

#### 3.2.1. Effects of TRPA1 in Animal Models of Colitis

The studies investigating the role of TRPA1 in animal models have reported heterogeneous outcomes depending on the specific colitis model employed. For instance, in DSS-induced colitis, TRPA1 modulation was associated with reduced inflammatory responses in most studies but with increased visceral sensitivity [22,24,27,28,29,30]. In TNBS- and DNBS-induced colitis, TRPA1 involvement was predominantly linked to exacerbation of nociceptive responses [25,31,32,33,34,35]. The OM-colitis model demonstrated a contribution of TRPA1 to neurogenic inflammation [36]. A functional antagonism between TRPA1 and TRPV1 was observed in the C57BL/6 mice administered intrarectal capsazepine; desensitization of TRPA1/TRPV1 decreased pain but also disrupted the intestinal epithelial barrier [26]. A further marked reduction in nociceptive responses was noted after combined inhibition of TRPA1 and TRPV1.

#### 3.2.2. Effects of TRPV1 in Animal Models of Colitis

Different functional outcomes of TRPV1 modulation were observed depending on the model used. Increased TRPV1 activity was generally associated with increased inflammation and visceral sensitivity in DSS-induced colitis [24,37,46,47,48]. The majority of research using the TNBS model found that TRPV1 activation resulted in worse outcomes, whereas antagonism reduced pain and inflammation [25,45,51,52,53,54,55]. In DNBS-induced colitis, TRPV1 antagonism was associated with reduced nociception [49,50]. The oxazolone-induced colitis model demonstrated a reduction in inflammation associated with TRPV1 activation [56]. In the intrarectal capsazepine model using C57BL/6 mice, combined TRPA1/TRPV1 desensitization alleviated pain responses but promoted colonic mucosal barrier damage [26]. The synergistic effect between TRPV1 and TRPV2 inhibition similarly led to reduced nociceptive sensitivity in TNBS models.

#### 3.2.3. Effects of TRPV2 in Animal Models of Colitis

Various model-dependent effects were indicated in TRPV2-related studies. Increased TRPV2 expression was associated with worsening colonic inflammation in the DSS-induced colitis model [57]. In the TNBS-induced colitis model, reduced TRPV2 activity correlated with decreased pain sensitivity [45]. Synergistic modulation involving TRPV1 and TRPV2 has also been reported to reduce nociceptive responses in TNBS-treated animals.

#### 3.2.4. Effects of TRPV4 in Animal Models of Colitis

In each of the experimental models considered, TRPV4 demonstrated a pro-nociceptive and pro-inflammatory profile. Specifically, in colitis caused by TNBS, elevated TRPV4 expression was linked to enhanced pain sensitivity and inflammation [38]. In DSS-induced colitis, TRPV4 upregulation correlated with elevated inflammatory responses [39,58].

#### 3.2.5. Effects of TRPM2 in Animal Models of Colitis

Depending on the animal colitis model used, different effects for TRPM2 were specified. Increased colonic inflammation was demonstrated to be associated with higher TRPM2 expression in the DSS-induced colitis model [60]. In models of TNBS-induced colitis, increased TRPM2 expression correlated with both heightened inflammation and enhanced pain sensitivity [59,61].

#### 3.2.6. Effects of TRPM3 in Animal Models of Colitis

The study by King et al. [62] utilized a DSS-induced colitis model and reported increased TRPM3 activity, which was associated with enhanced pain sensitivity.

#### 3.2.7. Effects of TRPM8 in Animal Models of Colitis

In the DSS and TNBS colitis models, heightened TRPM8 activity was consistently associated with increased pain sensitivity [63,65]. In addition, elevated TRPM8 expression demonstrated a positive correlation with both increased inflammation and greater pain sensitivity in animals with TNBS-induced colitis [64,66,67].

#### 3.2.8. Effects of TRPC6 in Animal Models of Colitis

The study by Nishiyama et al. [68] examined the role of TRPC6 in the DSS-induced colitis model. It demonstrated that increased TRPC6 activity was associated with a reduction in colonic inflammation [68].

**Figure 4:**
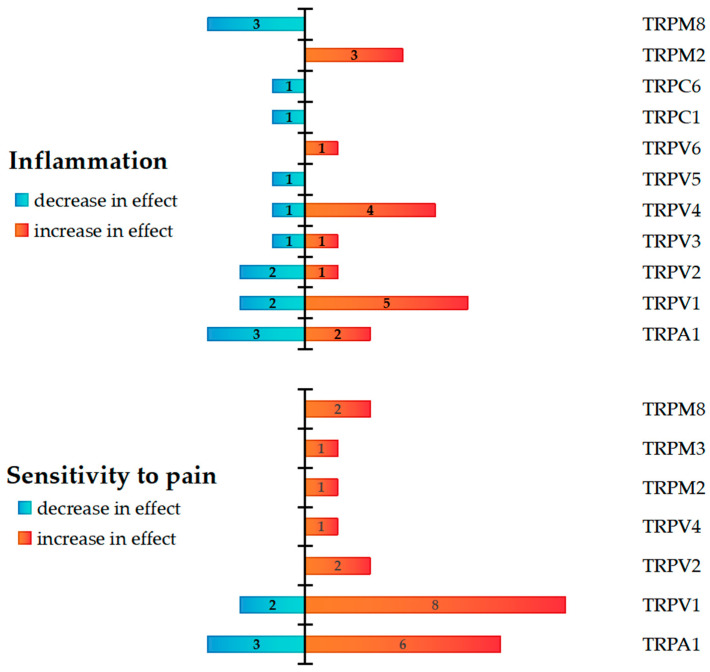
Quantitative data demonstrating effects related to pain sensitivity and inflammatory response in TRP-channeling studies, extracted from selected articles.

Focused on inflammation and pain sensitivity, the reviewed studies explored TRP channels, noting both potentiation and attenuation of the effects. The relevant findings from the selected articles were quantified for each channel, and the summarized information is presented in Figure 4. The authors’ one conclusion is interpreted as a single observation. The data presented are aggregated from both human and animal studies. The findings regarding the function of the channel were not summarized if the Materials and Methods section contained data from both humans and animals. Therefore, it was accepted that a single article could only show one outcome for a single channel.

A total of 58 observations were identified: 32 of these (55.2%) dealt with inflammation, and 15 of those cases (46.9%) showed a decrease in effect, while 17 cases (53.1%) showed an increase. In 26 observations, sensitivity to pain was measured (44.8%); in 5 cases, the effect decreased (19.2%), while in 21 cases, it increased (80.8%).

**Figure 5:**
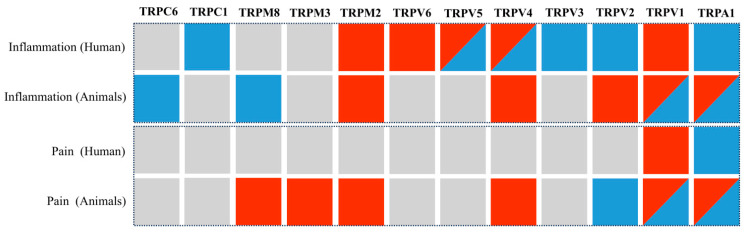
The role of TRP-channels in inflammation and pain: summarized data from human [5,22,23,37,38,39,40,42,43,44] and animal [22,24,25,26,27,28,29,30,31,32,33,34,35,36,37,38,39,45,46,47,48,49,50,51,52,53,54,55,56,57,58,59,60,61,62,63,64,65,66,67,68] studies. Blue: anti-inflammatory (protective) effects. Red: pro-inflammatory (exacerbating) effects. Gray: no data available.

Based on research conducted in both human (clinical) and animal (preclinical) models, Figure 5 summarizes the effects of TRP channels activity in the context of inflammation and pain.

A total of 48 distinct findings related to TRP channel activity were extracted from the included studies: 11 (22.9%) were only pro-inflammatory, 8 (16.7%) were solely anti-inflammatory, 6 (12.5%) showed both pro- and anti-inflammatory activity, and 23 (47.9%) had no data provided (Figure 5). Pro-inflammatory or pro-nociceptive effects were frequently associated with TRPV1, TRPA1, TRPV2, and TRPV4 in both human and animal studies. On the other hand, the main anti-inflammatory features of TRPV3, TRPC1, and TRPC6 were demonstrated. With no evidence of anti-inflammatory qualities, TRPM3, TRPM2, and TRPV6 were only associated with pro-inflammatory effects. Both pro- and anti-inflammatory effects have been demonstrated for TRPV1, TRPA1, TRPV4, and TRPV5, depending on the experimental context. Notably, the patterns varied between species and between settings of pain and inflammation (Figure 5).

## 4. Discussion

### 4.1. TRPA1

TRPA1 has been implicated in the pathogenesis of IBD and is frequently discussed alongside TRPV1 considering their comparable expression patterns and functional interactions in gastrointestinal inflammation [87]. TRPA1 is also considered a potential therapeutic target for the treatment of IBD-associated pain [26,88]. However, it is important to exercise caution when implementing such interventions in clinical settings due to their potential negative adverse effects.

Taken together, the data from both clinical and preclinical studies underscore the complex and context-dependent role of TRPA1 in the development of inflammation and pain associated with IBD. According to human research, TRPA1 is involved in the dynamic regulation of nociceptive and inflammatory signaling, as demonstrated by the increase in its expression throughout the active phase of the disease and changes in promoter methylation [22,23]. Specifically, TRPA1 may shift to an anti-inflammatory role during the active phase of inflammation, which may be influenced by the reduction in SP, NKA, NKB, and TNF-α. This observation suggests that TRPA1 functions as both a promoter of nociceptive signaling in various pathological conditions and a regulator of neurogenic inflammation through the suppression of important pro-inflammatory mediators [88,89].

Preclinical studies further corroborate this duality, although outcomes range greatly depending on the colitis model and the method employed for TRPA1 modulation. Elevated visceral sensitivity is generally associated with TRPA1 activation or expression in DNBS- and TNBS-induced colitis, whereas attenuation of the inflammatory markers is the frequent outcome of its genetic deletion or pharmacological inhibition [25,31,32,33,34,35]. However, in studies using DSS-induced colitis models, suppression of inflammation following TRPA1 inhibition or deletion is not consistently accompanied by changes in pain-related behavior, suggesting a dissociation between inflammatory and nociceptive outcomes [22,24,27,28,29,30]. Additionally, in the mustard oil (OM)-induced colitis model, TRPA1 has been implicated in the initiation of neurogenic inflammation [36]. These findings suggest that the pro-inflammatory and pro-nociceptive functions of this channel may be distinct depending on particular conditions. Moreover, studies on the co-modulation of TRPA1/TRPV1 additionally demonstrate their functional crosstalk and compensatory mechanisms in intestinal inflammation and barrier function [26]. In particular, combined desensitization of both channels reduces pain but may aggravate colon mucosal injury, emphasizing the necessity of preserving physiological TRP signaling for mucosal homeostasis. The observed antagonistic interplay between TRPA1 and TRPV1 may hold therapeutic potential, but its application requires precise modulation to avoid compromising epithelial integrity.

### 4.2. TRPV1-TRPV6

In the context of IBD, TRPV1 is frequently implicated in the pathogenesis of abdominal pain, VHS, and mucosal inflammation [87]. Increased TRPV1-positive nerve fibers are correlated with pain severity in quiescent CD and UC patients, which suggests that TRPV1 plays a predominantly pro-nociceptive and pro-inflammatory role in IBD [43]. This supports the hypothesis that TRPV1 significantly contributes to the development of persistent abdominal pain and IBS-like symptoms, even during remission stages [8]. However, the variable gene expression patterns imply a multifaceted control system: a decrease in TRPV1 mRNA during acute inflammatory phases might indicate inhibitory feedback mechanisms or specific degradation, whereas heightened expression during quiescent periods could suggest a reparative or adaptive response [22,42]. The discrepancy between mRNA and protein expression levels highlights potential post-transcriptional and translational regulatory controls, including microRNA-mediated silencing, mRNA stability variations, or altered protein turnover under inflammatory conditions. Differential expression of TRPV1 in epithelial versus neuronal cells points to distinct, possibly cell-specific, functions. In epithelial cells, TRPV1 may contribute to barrier signaling and cross-talk with other TRP channels, like TRPV3 [40]. These functions demonstrate a multifaceted role of TRPV1 beyond nociception, including modulation of epithelial integrity and immune responses. Preclinical evidence strongly demonstrates TRPV1’s pro-inflammatory and pro-nociceptive role in intestinal inflammation. Symptom relief by pharmacological antagonism suggests that TRPV1 is a key mediator of pain and inflammatory responses in the DSS-induced colitis model, highlighting its potential as a therapeutic target [90]. Similarly, in TNBS-induced colitis, activation of TRPV1 aggravates inflammatory and nociceptive responses, while its antagonism or genetic deletion reduces symptom severity [25,45,51,52,53,54,55]. Selective TRPV1 inhibition reduces nociceptive sensitivity in DNBS-induced colitis, confirming its pro-nociceptive role [49,50]. However, the variability between models indicates that TRPV1’s function may be influenced by distinct inflammatory pathways and cellular environments specific to each model. Contrastingly, the oxazolone model reveals an anti-inflammatory role for TRPV1, highlighting the context-dependent duality of the channel [56]. Changes in immune polarization (Th1/Th17 dominance in TNBS/DNBS versus Th2 with oxazolone) or differences in TRPV1 expression in neuronal versus non-neuronal cells may influence downstream signaling. Importantly, intrarectal administration of the TRPV1 antagonist capsazepine in C57BL/6 mice reduces pain responses; however, it may potentially compromise mucosal barrier integrity [26]. This finding illustrates the complex balance between alleviating nociception and maintaining epithelial homeostasis when targeting TRPV1 locally within the colon. Moreover, combined desensitization of TRPV1 and TRPA1 channels reduces pain behaviors but compromises mucosal defenses, emphasizing the intricacies of targeting multiple TRP channels simultaneously [91]. Synergistic inhibition of TRPV1 and TRPV2 further decreases nociceptive responses, suggesting functional crosstalk among TRP family members that modulates both inflammation and pain signaling.

TRPV2 is a sensor of harmful heat, but subsequent studies have revealed a more complex role for TRPV2 that extends beyond temperature perception [92]. Specifically, TRPV2 has been identified as a key candidate cancer biomarker and potential therapeutic target, as well as a significant modulator of IBD pathophysiology [93]. Based on clinical data, there is a negative correlation between disease activity and TRPV2 mRNA expression in PBMCs from patients with Crohn’s disease and ulcerative colitis [5]. This suggests that the suppression of its expression may be a compensatory mechanism to limit excessive inflammation. Reduced expression of the TRPV2 gene in colonic tissues from UC patients during both active and remission periods lends credence to this notion [41].

Preclinical studies provide further complexity. Enhanced colonic inflammation has been associated with increased TRPV2 expression in DSS-induced colitis [57], suggesting a pro-inflammatory role. Conversely, TNBS-induced colitis studies demonstrate that reduced TRPV2 activity correlates with attenuated pain sensitivity [45], suggesting TRPV2’s involvement in nociceptive pathways. The reported synergistic modulation between TRPV1 and TRPV2 reducing nociceptive responses in TNBS models points toward functional crosstalk within the TRP channel family, potentially allowing fine-tuning of pain and inflammatory signaling. Data from animal studies confirm that the channel may contribute to visceral nociception and, when overexpressed, enhance mucous membrane inflammation. The reasons for this dichotomy are still unclear, but could be related to different channel activation states, interacting partners, or intracellular signaling cascades that are specific to cell types and disease stages. TRPV3 expression in clinical studies predominantly demonstrates a trend toward downregulation in active IBD, suggesting a potential protective or modulatory role in disease activity [5]. This inverse correlation may indicate that TRPV3 contributes to the maintenance of mucosal integrity and modulates inflammation, whereas decreased expression exacerbates pathological processes. However, some studies found no significant difference in TRPV3 expression in patients with UC compared with controls, highlighting potential variability due to differences in patient cohorts, disease stage, or study methodology [40]. Additionally, positive correlations between TRPV3 and other TRP channels, such as TRPV1 and TRPV4, may indicate their potential synergy providing unidirectional or multidirectional effects in nociception and epithelial signaling during inflammation.

Clinical data consistently indicate that TRPV4 plays a predominantly pro-inflammatory and pro-nociceptive role in IBD pathophysiology. Thus, elevated TRPV4 mRNA expression in PBMCs of CD patients [5,38] and increased protein levels in the colonic epithelium of UC patients [40] underscore its active involvement in perpetuating intestinal inflammation. However, one study has demonstrated that TRPV4 gene expression was higher in remission UC patients compared to active UC, and protein expression was elevated in all intestinal layers of controls relative to UC patients [41]. This suggests a complex, possibly context-dependent role of TRPV4, where its expression may be involved not only in active inflammation but also in mucosal homeostasis or repair processes.

Preclinical data further suggest mainly the pro-inflammatory and pro-nociceptive role of TRPV4. Both DSS- and TNBS-induced colitis studies show that increased TRPV4 expression correlates with enhanced inflammation and pain sensitivity [38,39,58]. These findings reinforce the notion that TRPV4 contributes to intestinal inflammation and nociception, possibly through mechanisms similar to those observed in human epithelial cells. Nevertheless, the apparent paradox between increased TRPV4 expression in remission phases and its pro-inflammatory role during active disease might reflect a dual function: TRPV4 could participate in normal epithelial signaling and barrier integrity under physiological conditions but exacerbate inflammation when dysregulated. Alternatively, post-transcriptional modifications or cell-type-specific expression patterns might underlie this divergence.

Despite the growing interest in the role of TRPV channels in IBD, studies directly examining TRPV5 function in UC and CD pathogenesis are relatively limited. A study by Toledo Mauriño et al. [41] represents a substantial advancement in comprehending the role of TRPV5 in UC. This multi-channel human study has demonstrated an inverse relationship between TRPV5 expression and the active inflammatory state in UC, thus suggesting that downregulation of both TRPV5 mRNA and protein expression in active UC may contribute to the development or exacerbation of the disease. The potential explanations for said correlation include disrupted calcium homeostasis in intestinal epithelial cells, which may contribute to inflammation and tissue damage; modulation of local immune responses in the gut, promoting pro-inflammatory cytokine production or impairing anti-inflammatory mechanisms; and impaired barrier function followed by increased intestinal permeability, compromised intestinal barrier integrity, and chronic tissue damage seen in UC.

Given the evidence of shared inflammatory pathways between UC and CD, it can be hypothesized that TRPV5 involvement in the pathogenesis of CD is also possible. TRPV6 is highly expressed in the gastrointestinal tract, specifically in the intestinal epithelium, where it may influence intestinal barrier permeability and cellular signaling. TRPV6 is known to play a pivotal role in transcellular Ca^2+^ transport, intracellular Ca^2+^ reuptake, and local Ca^2+^ regulation maintaining a low concentration of this ion in the cellular environment of different tissues [11]. A recent study [41] has provided novel insights into the potential involvement of TRPV6 in IBD. It focused on the analysis of TRPV6 expression in colon tissue of UC patients. The elevated TRPV6 gene and protein expression levels in the samples from active UC patients strongly implicate this channel in the disease activity and progression. It is hypothesized that the potential mechanisms may include dysregulated calcium homeostasis due to excessive calcium entry into intestinal epithelial cells, compromised intestinal epithelial barrier function, or modulation of inflammatory signaling since TRPV6-mediated calcium influx could potentially activate pro-inflammatory pathways within the colonic epithelium resulting in an excessive inflammatory response. Given the common pathogenetic mechanisms underlying both UC and CD, such as disruption of the intestinal barrier, dysregulation of the immune response and genetic predisposition, it is conceivable that TRPV6 may also contribute to the pathogenesis of CD. Interestingly, despite high structural and functional similarity, TRPV5 and TRPV6 appear to have different effects in IBD (predominantly an anti-inflammatory role in TRPV5 and a pro-inflammatory role in TRPV6). Possible explanations may include different localization (TRPV5 is highly expressed in the kidneys and TRPV6 in the intestine [72]), involvement in separate signaling pathways, regulation by various factors, and interplay with interacting proteins resulting in diverse functional consequences.

### 4.3. TRPM2, TRPM3, and TRPM8

Recent investigations have explored the contribution of TRPM2 to IBD, utilizing both human patient samples and animal models of colitis. A study by Morita et al. [5] focused on the analysis of PBMCs from UC and CD patients, revealing elevated TRPM2 mRNA expression compared to healthy controls. This finding suggests a potential pro-inflammatory role for TRPM2 in IBD progression, possibly contributing to intestinal inflammation. Animal models of colitis, specifically TNBS [59,61] and DSS-induced models [60], corroborate this pro-inflammatory role and also reveal a potential link between TRPM2 and enhanced pain sensitivity in TNBS-induced colitis. This finding is particularly noteworthy as it suggests that TRPM2 is involved not only in the inflammatory pathology but also in the VHS that frequently accompanies IBD. TRPM2 upregulation in IBD and experimental colitis can be associated with its activation by reactive oxygen species (ROS), which are significantly elevated during inflammation, subsequently resulting in an exacerbated inflammatory response. Furthermore, TRPM2 can modulate activation and proliferation of immune cells, and cytokine production, thus contributing to the dysregulated immune pattern in IBD.

King et al. [62] has raised questions about TRPM3 involvement in colonic sensation during colitis. The most significant result from this study is the association of increased TRPM3 activity with enhanced colonic sensitivity in a DSS-induced colitis model. Since pain and VHS are frequent complaints in IBD patients, this observation is quite crucial. However, the obtained data are limited to an animal model, thus further research is required to elucidate the therapeutic potential of TRPM3 in IBD patients.

There is emerging evidence that TRPM8 is involved in the gastrointestinal physiology and pathophysiology [76]. Although clinical studies investigating TRPM8 in IBD are currently lacking, preclinical data from animal models provide insight into its potential role in intestinal inflammation and pain. It is possible that clinical data do exist. However, they were either excluded from the present systematic review due to not meeting inclusion criteria or were not retrieved in the conducted literature searches. Preclinical data utilizing DSS- and TNBS-induced colitis consistently report increased TRPM8 activity associated with heightened pain sensitivity [63,65]. Interestingly, activation of TRPM8 in these models is also linked to attenuated inflammatory responses, suggesting a dual modulatory function [64,66,67]. Activation of TRPM8 appears to initiate signaling pathways that suppress the production of pro-inflammatory mediators, which may underlie TRPM8 protective effect against mucosal injury. For instance, TRPM8-mediated protection against indomethacin-induced small intestinal damage occurs via CGRP release, highlighting its role in neuroimmune modulation within the gut [15]. These findings suggest that TRPM8 may exert an anti-inflammatory and analgesic influence in the context of experimental colitis, contrasting with the predominantly pro-inflammatory profile observed for other TRP channels.

### 4.4. TRPC1 and TRPC6

In the current systematic review, selected articles examine the role of TRPC1 and TRPC6 channels. Literature data suggest that they are associated with inflammation in mice with DSS-induced colitis, as well as in patients with CD and UC [5,68]. Morita et al. indicated that TRPC1 mRNA expression was reduced in PBMCs from both UC and CD patients compared to healthy controls [5]. Another study has considered the relationship between TRPC6 channel activation and prevention of colitis progression [68], demonstrating its protective role.

TRPC1 is a substrate of caspase-11, which controls inflammatory responses, and this channel degradation increases interleukin-1β (IL-1β) production in macrophages [94]. It is well established that abnormalities in TRPC1 expression increase macrophage death or caspase-1-independent IL-1β release. TRPC1^(−/−)^ mice exhibit reduced lifespan, lung tissue damage, and systemic infection due to impaired activation of the Toll-like receptor 4 (TLR4)-TRPC1 pathway [95]. This pathway is often triggered by lipopolysaccharide (LPS), which is known to cause transcriptional alterations that inhibit the synthesis of pro-inflammatory cytokines [96]. Furthermore, it is known that TRPC1-mediated Ca^2+^ influx is responsible for macrophage polarization toward the M1 inflammatory phenotype [16,96]. In a mouse model of antigen-mediated anaphylaxis, TRPC1 promoted recovery by suppressing TNF-α release from mast cells [17]. Nonetheless, the activity of these channels seemingly pertains to more than just immune cells. A deficit in endothelial TRPC1 aggravates metabolic problems associated with obesity, whereas its augmented expression provides a marked safeguarding effect [97]. Heteromeric TRPC1/4 expressed in the pulmonary endothelium may play a protective role. The microvascular endothelium’s permeability may rise as a result of the Ca^2+^ inflow, which could lead to an increase in inflammation. However, the protective role is achieved by preserving autophagy in cases of hypoxic pulmonary arterial hypertension [95]. High TRPC1 expression in ROS-induced human A549 lung epithelial cell line resulted in increased expression of oxidative, fibrotic, inflammatory and apoptotic genes [98]. Research on isolated mouse heart muscle cells, within a model of LPS-induced cardiac dysfunction, demonstrated that knockout of Trpc1 or Trpc6 prevented Ca^2+^ leakage from the sarcoplasmic or endoplasmic reticulum, thereby enhancing survival rates [99]. The effect of TRPC-mediated Ca^2+^ influx appears to be dependent on the particular physiological or pathological context, according to mounting data.

In contrast to TRPC1, which is extensively expressed, TRPC6 resides in tissues containing smooth muscle cells, including the myometrium, stomach, colon, lung, and blood vessels. Through activation by receptor tyrosine kinases or diacylglycerol (DAG), TRPC6 modulates immune cell responses and smooth muscle contractility [100]. Given the role of these channels in muscle cell function, TRPC6 activation in intestinal smooth muscle may be a factor contributing to stool changes and abdominal discomfort in IBD. However, this idea requires further comprehensive study. TRPC6 channels are reported to permeate metal ions such as Zn^2+^ and Fe^2+^. In a study aimed to determine the role of TRPC6-mediated Zn^2+^ influx in intestinal stress resilience, Nishiyama K et al. [68] found that TRPC6 channel was associated with DSS-induced colitis in mice. Reduced colonic Zn^2+^ levels, elevated mRNA for the antioxidant proteins, and decreased microbiota diversity were the outcomes of TRPC6 deficiency in this investigation. The findings of this study confirmed previously held beliefs regarding the role of Zn^2+^ in maintaining gut homeostasis [101]. In IBD, it is well known that the body’s zinc levels fall, impairing the gastrointestinal epithelium’s ability to function. This condition also impacts numerous immune cells, increasing T and B lymphocytes’ proliferative response to interleukin-6 (IL-6) and interleukin-2 (IL-2) stimulation. Additionally, it has been found that inflammatory cytokines including TNF-α and IL-1β are elevated when Zn^2+^ concentrations fall [102,103]. Moreover, in a study by Nishiyama K. et al. [68], the protective role of TRPC6 was confirmed using its activator (PPZ2). It has been shown that PPZ2 treatment prevented the progression of DSS-induced colitis and also significantly suppressed IL-6 expression in the colon. As a result, raising intestinal Zn^2+^ level via TRPC6 channel activation may be a potential future therapeutic approach for IBD. Since the data in the selected articles were obtained either from animal models or from humans but using only the mRNA detection method, the role of TRPC1 and TRPC6 channels in colitis and IBD remains unclear. Nevertheless, the data already collected show that the expression levels of these channels can be considered promising markers of IBD.

**Figure 6:**
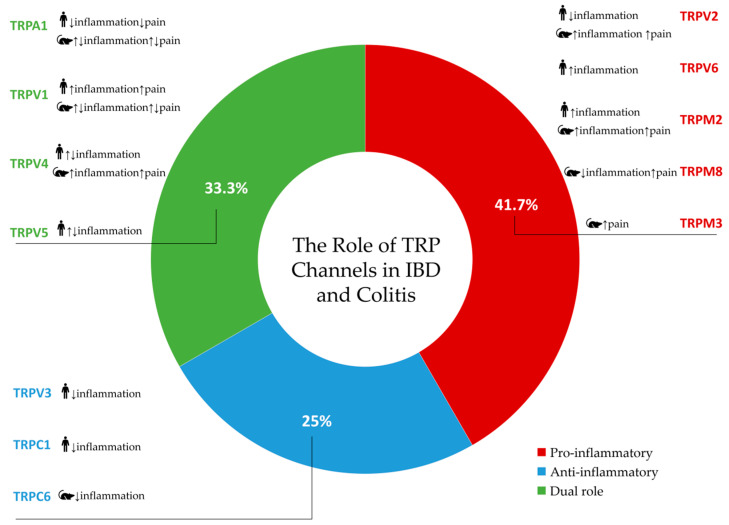
The relative distribution of anti-inflammatory, pro-inflammatory, and dual roles of TRP channels in IBD and colitis. An upward arrow (↑) indicates an increase in effect, while a downward arrow (↓) indicates a decrease in effect.

Overall, the evidence gathered in this systematic review demonstrates that TRP channels display distinct and sometimes contradictory roles in the regulation of inflammation and nociception in IBD and experimental colitis. A circular diagram summarizing the findings of this systematic review, including the categories of studies and the identified effects in the selected studies, is presented in Figure 6.

## 5. Limitations of the Study

This systematic review has several important limitations that should be acknowledged. First, the protocol of the systematic review was not registered in a public database (e.g., PROSPERO), which may increase the risk of reporting bias and reduce methodological transparency. Second, the overall quality of evidence was low: most human studies were judged as very low quality, while the majority of animal studies exhibited an unclear risk of bias, particularly due to insufficient reporting of randomization and blinding procedures. Consequently, the strength of the conclusions is limited, and the findings should be interpreted with caution. Third, a substantial proportion of the included studies were conducted exclusively in either human or animal models, thereby restricting the possibility of direct cross-species comparisons and limiting the translational value of the results. Fourth, heterogeneity in study design, experimental models, outcome measures, and analytical approaches further complicated data synthesis. In addition, many studies were constrained by small sample sizes and conflicting data, which may further compromise the reliability and interpretation of the results.

Taken together, these factors highlight the necessity for well-designed, adequately powered, and transparently reported studies in both human and animal models to enable robust conclusions regarding the role of TRP channels in IBD pathogenesis and their potential as therapeutic targets.

## 6. Conclusions

This systematic review synthesizes clinical and preclinical data on the involvement of TRP channels in the pathogenesis of IBD, highlighting their multifaceted and context-dependent roles in modulating inflammation and visceral sensitivity. The TRPA1, TRPV1, TRPV4, and TRPV5 channels exhibit dual, often opposing, pro- and anti-inflammatory effects depending on the disease stage, the experimental model, and the cellular localization, while consistently contributing to visceral pain signaling. TRPM2, TRPM3, and TRPM8 are predominantly associated with enhanced nociception. Furthermore, the TRPV3, TRPC1, and TRPC6 channels demonstrate primarily anti-inflammatory properties, whereas TRPV6, TRPM2, and TRPM3 manifest pro-inflammatory activity. The observed heterogeneity emphasizes the complex role of TRP channels involved in IBD pathophysiology and the necessity of precise, context-specific modulation. A better understanding of channel-specific mechanisms has the potential to facilitate the development of targeted therapeutic strategies aimed at controlling inflammation and alleviating pain.

## Figures and Tables

**Table 1:** Features of the included human studies.

TRP Channel	Category of Studies	Author, Year	Confirmed and/or Expected Effects	Conclusion
TRPA1	Human + animal multi-channel studies	Kun J. et al., 2014 [22]	TRPA1 expression is significantly increased in patients with active IBD, but not inactive IBD, compared with non-inflamed samples	Anti-inflammatory role in active IBD and reduction in pain sensitivity
	Human single-channel studies	Gombert S. et al., 2019 [23]	Increased TRPA1 promoter methylation correlates with dysregulated TRPA1 expression and enhanced peripheral pain sensitivity in CD patients	
TRPV1	Human + animal multi-channel studies	Kun J. et al., 2014 [22]	TRPV1 mRNA is significantly decreased in patients with active IBD compared to non-inflamed group	Mainly pro-inflammatory role and increased pain sensitivity
	Human single-channel studies	Luo C. et al., 2017 [44]	TRPV1 immunoreactivity was highly expressed on epithelial cells and infiltrating inflammatory cells in colon biopsies from patients with active IBD	
	Human single-channel studies	Akbar A. et al., 2010 [43]	Abdominal pain and visceral hypersensitivity	
	Human single-channel studies	Toledo-Mauriño et al., 2018 [42]	Increased TRPV1 gene expression in remission UC patients compared to active UC patients; higher TRPV1 protein expression observed in all intestinal layers of active UC patients compared to non-IBD controls	
	Human multi-channel studies	Rizopoulos T., 2018 [40]	Statistically decreased TRPV1 expression levels were demonstrated for patients with active UC compared to the control group	
	Human + animal single-channel studies	Duo L. et al., 2020 [37]	TRPV1 is highly expressed in patients with IBD	
TRPV2	Human multi-channel studies	Morita T. et al., 2020 [5]	TRPV2 mRNA expression was negatively correlated with leukocyte count in UC; decreased TRPV2 mRNA expression levels in PBMCs of both UC and CD patients which negatively correlated with disease activity in both groups, suggesting a potential role in modulating inflammation	Anti-inflammatory role
	Human multi-channel studies	Toledo Mauriño et al., 2020 [41]	The inner layers of the intestine had increased expression of TRPV2; TRPV2 gene expression was lower in samples of active and remission UC patients compared to control group; TRPV2 protein expression was upregulated in the mucosa and submucosa but lower in the muscular layer and serosa cells of controls compared to UC patients	
TRPV3	Human multi-channel studies	Morita T. et al., 2020 [5]	TRPV3 expression was reduced in patients with CD	Anti-inflammatory role
	Human multi-channel studies	Rizopoulos T., 2018 [40]	No significant difference for TRPV3 expression levels between UC and control samples	
	Human multi-channel studies	Toledo Mauriño et al., 2020 [41]	TRPV3 gene and protein expression was higher in controls than in active UC patients, suggesting downregulated TRPV3 expression to be associated with disease activity	
TRPV4	Human multi-channel studies	Morita T. et al., 2020 [5]	Heightened TRPV4 mRNA expression levels in PBMCs of CD patients compared to healthy controls, positive correlation of its mRNA expression with the serum albumin level in the UC group and with the CRP level in the CD group	Pro- and anti-inflammatory roles
	Human + animal single-channel studies	Fichna J. et al., 2012 [38]	TRPV4 mRNA expression was significantly elevated in patients with CD and UC compared with healthy subjects (2.9 and 4.5-fold, respectively)	
	Human multi-channel studies	Rizopoulos T., 2018 [40]	TRPV4 expression levels were significantly increased in the colonic epithelium of UC patients compared to non-IBD controls	
	Human multi-channel studies	Toledo Mauriño et al., 2020 [41]	TRPV4 expression in UC patients in remission and control groups was increased compared to active UC; high TRPV4 expression may be associated with a healthy colon	
	Human + animal single-channel studies	D’Aldebert E. et al., 2011 [39]	TRPV4 activation in Caco-2 and human colon epithelial cells increased Ca^2+^ and chemokine release, supporting a pro-inflammatory epithelial phenotype	
TRPV5	Human multi-channel studies	Toledo Mauriño et al., 2020 [41]	The absence of TRPV5 appears to correlate with UC induction	Anti-inflammatory role if this channel is activated, and pro-inflammatory role if this channel is inhibited
TRPV6	Human multi-channel studies	Toledo Mauriño et al., 2020 [41]	TRPV6 is highly expressed in all layers of the intestine in patients with UC, and this appears to be clearly associated with disease activity	Pro-inflammatory role
TRPM2	Human multi-channel studies	Morita T. et al., 2020 [5]	Increased expression levels in PBMCs from UC and CD patients	Pro-inflammatory role
TRPC1	Human multi-channel studies	Morita T. et al., 2020 [5]	Reduced expression in PBMCs from UC and CD patients may enhance disease progression	Anti-inflammatory role

**TRP:** Transient receptor potential channel; **IBD:** Inflammatory Bowel Disease; **CD:** Crohn’s disease; **UC:** Ulcerative colitis; **PBMCs:** Peripheral blood mononuclear cells; **CRP:** C-reactive protein.

**Table 2:** Features of the included animal studies.

TRP Channel	Category of Studies	Author, Year	Model	Confirmed and/or Expected Effects	Conclusion
TRPA1	Human + animal multi-channel studies	Kun J. et al., 2014 [22]	DSS-induced colitis in mice	Protective role in colitis	Pro- and anti-inflammatory roles with pain sensitivity increase
	Animal single-channel studies	Kistner K. et al., 2016 [27]	DSS-induced colitis in mice	Desensitization of TRPA1 attenuates neurogenic inflammation	
	Animal single-channel studies	Mitrovic M. et al., 2010 [28]	DSS-induced colitis in mice	Visceral hypersensitivity mediated by TRPA1 agonist AITC	
	Animal single-channel studies	Jain P. et al., 2020 [29]	DSS-induced colitis in mice	TRPA1 contributes to colitis-associated mechanical hypersensitivity via increased expression and activity in DRG neurons, with its blockade reducing hypersensitivity without affecting colitis severity	
	Animal single-channel studies	Yang Y. et al., 2019 [30]	DSS-induced colitis in mice and rats	In DSS-induced colitis, TRPA1 expression is upregulated, and TRPA1 activation exacerbates abnormal colonic motility; pharmacological or genetic inhibition of TRPA1 alleviates these motility disturbances	
	Animal multi-channel studies	Utsumi D. et al., 2018 [24]	DSS-induced colitis in mice	TRPV1 and TRPA1 expression in sensory neurons plays a critical role in the progression of colonic inflammation in DSS-induced colitis in mice	
	Animal single-channel studies	Chen J., 2013 [31]	DNBS-induced colitis in rats	Increased visceral hypersensitivity; TRPA1 antagonism mitigates these effect	
	Animal single-channel studies	Kurahara L. H. et al., 2018 [32]	TNBS-induced colitis in mice	In TRPA1 knockout mice, the extent of inflammation and fibrosis is more pronounced compared to wild-type mice	
	Animal multi-channel studies	Vermeulen W. et al., 2013 [25]	TNBS-induced colitis in rats	Visceral hypersensitivity	
	Animal single-channel studies	Li Q. et al., 2016 [33]	TNBS-induced colitis in rats	Visceral hypersensitivity	
	Animal single-channel studies	Brierley S. M. et al., 2009 [34]	TNBS-induced colitis in mice	Visceral hypersensitivity	
	Animal single-channel studies	Cattaruzza F. et al., 2010 [35]	TNBS-induced colitis in mice	Visceral hypersensitivity	
	Animal multi-channel studies	Kumar V. et al., 2022 [26]	C57BL/6 mice treated with intrarectal capsazepine	Damaged mucosa, increased intestinal permeability	
	Animal single-channel studies	Kimball E. S. et al., 2007 [36]	OM colitis in mice	Increased mRNA levels of various neuropeptides and mediators associated with pain and inflammation	
TRPV1	Human + animal single-channel studies	Duo L. et al., 2020 [37]	DSS-induced colitis in mice	Enhanced Th17 cell differentiation and dendritic cell-mediated inflammation	Pro-inflammatory role with pain sensitivity increase, but only antagonism reduces inflammation and pain
	Animal single-channel studies	Lapointe T. K. et al., 2015 [46]	DSS-induced colitis in mice	Increased inflammation, increased release of CGRP and SP, visceral hypersensitivity and pain-related behavior	
	Animal single-channel studies	Engel M. A. et al., 2012 [47]	DSS-induced colitis in mice	Increased inflammation, increased release of CGRP and SP	
	Animal multi-channel studies	Utsumi D. et al., 2018 [24]	DSS-induced colitis in mice	TRPV1 and TRPA1 expression in sensory neurons plays a critical role in the progression of colonic inflammation in DSS-induced colitis in mice	
	Animal single-channel studies	Matsumoto K. et al., 2012 [48]	DSS-induced colitis in rats	DSS-induced colitis leads to increased TRPV1 and 5-HT_3_ receptor expression and decreased 5-HT_4_ receptor expression in colonic mucosa, contributing to visceral hypersensitivity	
	Animal single-channel studies	Massa F. et al., 2006 [49]	DNBS-induced colitis in mice	Modulating of sensory pathways involved in colonic inflammation, possible protective effect	
	Animal single-channel studies	Mazor Y. et al., 2024 [50]	DNBS-induced colitis in rats	TRPV1 antagonism reduces pain and inflammation	
	Animal multi-channel studies	Vermeulen W. et al., 2013 [25]	TNBS-induced colitis in rats	Visceral hypersensitivity	
	Animal multi-channel studies	Matsumoto K. et al., 2023 [45]	TNBS-induced colitis in rats	Visceral hypersensitivity	
	Animal single-channel studies	De Schepper H. U. et al., 2008 [51]	TNBS-induced colitis in rats	TRPV1 receptor activation mediates afferent nerve sensitization during colitis-induced motility disorders in rats. Inhibition of TRPV1 signaling reduces colitis-induced motility disorders and afferent nerve sensitization	
	Animal single-channel studies	De Schepper H. U. et al., 2008 (2) [52]	TNBS-induced colitis in rats	TRPV1 receptors on unmyelinated C-fibers mediate colitis-induced sensitization of pelvic afferent nerve fibers in rats. Inhibition of TRPV1 signaling reduces colitis-induced sensitization and associated pain	
	Animal single-channel studies	Miranda A. et al., 2007 [53]	TNBS-induced colitis in rats	Increased visceral sensitivity; TRPV1 antagonism reduces both inflammation and hypersensitivity	
	Animal single-channel studies	Shen S. et al., 2017 [54]	TNBS-induced colitis in rats	Visceral hypersensitivity	
	Animal single-channel studies	Wu Y. et al., 2019 [55]	TNBS-induced colitis in mice	TLR4 signaling contributes to TRPV1 upregulation and peripheral sensitization in inflammatory conditions	
	Animal single-channel studies	Lee J. et al., 2012 [56]	oxazolone-induced colitis in mice	Excessive neutrophil accumulation; a protective role of TRPV1 expressing extrinsic sensory neurons in oxazolone induced colitis	
	Animal multi-channel studies	Kumar V. et al., 2022 [26]	C57BL/6 mice treated with intrarectal capsazepine	Damaged mucosa, increased intestinal permeability	
TRPV2	Animal multi-channel studies	Matsumoto K. et al., 2023 [45]	TNBS-induced colitis in rats	Visceral hypersensitivity	Pro-inflammatory role and increased pain sensitivity
	Animal single-channel studies	Issa C. M. et al., 2014 [57]	DSS-induced colitis in mice	Increased inflammation	
TRPV4	Human + animal single-channel studies	Fichna J. et al., 2012 [38]	TNBS-induced colitis in mice	Intestinal inflammation and colitis-associated pain	Pro-inflammatory role and increased pain sensitivity
	Human + animal single-channel studies	D’Aldebert E. et al., 2011 [39]	DSS-induced colitis in mice	TRPV4 mRNA expression was up-regulated when compared with control naïve tissues	
	Animal single-channel studies	Matsumoto K. et al., 2018 [58]	DSS-induced colitis in mice	Pro-inflammatory effects	
TRPM2	Animal single-channel studies	Matsumoto K. et al., 2016 [59]	TNBS-induced colitis in mice and rats	Increased visceromotor reflexes caused by balloon pressure, visceral hypersensitivity	Pro-inflammatory role and increased pain sensitivity
	Animal single-channel studies	Yamamoto S. et al., 2008 [60]	DSS-induced colitis in mice	Progression of colitis through its possible implication in oxidative stress signaling	
	Animal single-channel studies	Nakamoto T. et al., 2024 [61]	TNBS-induced colitis in mice	TRPM2 contributes to inflammation via Th1/Th17 pathways; TRPM2 mediates ROS-induced cytokine release and MAPK activation	
TRPM3	Animal single-channel studies	King J. W. et al., 2024 [62]	DSS-induced colitis in mice	Perception of noxious stimuli in colitis, colonic hypersensitivity	Increased pain sensitivity
TRPM8	Animal single-channel studies	Hosoya T. et al., 2014 [63]	TNBS-induced colitis in mice DSS-induced colitis in mice	Visceral hyperalgesia	Anti-inflammatory role, but with increased pain sensitivity
	Animal single-channel studies	Ramachandran R. et al., 2013 [64]	DSS-induced colitis in mice	Anti-inflammatory role of TRPM8 activation, partly mediated by inhibition of neuropeptide release	
	Animal single-channel studies	de Jong P. R. et al., 2015 [65]	TNBS-induced colitis in mice DSS-induced colitis in mice	TRPM8 deficiency leads to increased susceptibility to colitis, while CGRP administration ameliorates inflammation	
	Animal single-channel studies	Khalil M. et al., 2016 [66]	DSS-induced colitis in mice	TRPM8 deficiency leads to increased colitis severity, while activation of TRPM8 with menthol enemas provides protection	
	Animal single-channel studies	Zhang Z. et al., 2024 [67]	DSS-induced colitis in mice	The activation of TRPM8 attenuated DSS-induced colitis in mice	
TRPC6	Animal single-channel studies	Nishiyama K. et al., 2024 [68]	DSS-induced colitis in mice	TRPC6 expression increased in DSS-induced colitis; treatment with TRPC6 activator PPZ2 prevented DSS-induced colitis progression	Anti-inflammatory role

**TRP:** Transient receptor potential channel; **DSS:** Dextran Sulfate Sodium; **DNBS:** Dinitrobenzene sulfonic acid; **TNBS:** 2,4,6-Trinitrobenzene sulfonic acid; **OM:** Oil of mustard; **AITC:** Allyl isothiocyanate; **DRG:** Dorsal root ganglia neurons; **CGRP**: Calcitonin Gene-Related Peptide; **SP:** Substance P; **5-HT_3_ receptor:** 5-hydroxytryptamine_3_ receptor; **5-HT_4_ receptor:** 5-hydroxytryptamine_4_ receptor; **MAPK:** Mitogen-activated protein kinase expression and decreased; **Th1/Th17:** T helper 1/T helper 17; **ROS:** Reactive oxygen species.

## Data Availability

Data is contained within the article.

## Supplementary Materials

The following supporting information can be downloaded at https://www.mdpi.com/article/10.3390/ijms26199390/s1.

## Abbreviations

The following abbreviations are used in this manuscript:

TRP	Transient receptor potential channel
IBD	Inflammatory Bowel Disease
CD	Crohn’s disease
UC	Ulcerative colitis
GI	Gastrointestinal tract
IBS	Irritable bowel syndrome-like
VHS	Visceral hypersensitivity
PICO	Population, Intervention, Comparison, Outcome
PBMCs	Peripheral blood mononuclear cells
SP	Substance P
NKA	Neurokinin A
NKB	Neurokinin B
TNF-α	Tumor necrosis factor alpha
TRPV1	Transient receptor potential vanilloid 1
TRPV2	Transient receptor potential vanilloid 2
TRPV3	Transient receptor potential vanilloid 3
TRPV4	Transient receptor potential vanilloid 4
TRPV5	Transient receptor potential vanilloid 5
TRPV6	Transient receptor potential vanilloid 6
TRPA1	Transient receptor potential ankyrin 1
TRPM2	Transient receptor potential melastatin 2
TRPM3	Transient receptor potential melastatin 3
TRPM8	Transient receptor potential melastatin 8
TRPC1	Transient receptor potential canonical 1
TRPC6	Transient receptor potential canonical 6
IL	Interleukin
DAG	Diacylglycerol
CRP	C-reactive protein
4αPDD	4α-phorbol-12,13-didecanoate
DSS	Dextran Sulfate Sodium
DNBS	Dinitrobenzene sulfonic acid
TNBS	2,4,6-Trinitrobenzene sulfonic acid
OM	Oil of mustard
CGRP	Calcitonin Gene-Related Peptide
AITC	Allyl isothiocyanate
LPS	Lipopolysaccharide
MAPK	Mitogen-activated protein kinase
TLR4	Toll-like receptor 4
SPF	Specific pathogen free status
ROS	Reactive oxygen species
5-ASA	5-aminosalicylic acid
GPRs	G protein-coupled receptors
