# Supplementary material for: The Role of TRP Channels in Colitis and Inflammatory Bowel Disease: A Systematic Review
Source: Int J Mol Sci. 2025 Sep 25;26(19):9390. doi: 10.3390/ijms26199390 (PMC12524589; doi:10.3390/ijms26199390)
Supplement: Supplementary file 1 [file ijms-26-09390-s001.zip › ijms-3852071-supplementary.pdf]

PRISMA 2020 Main Checklist

**TITLE**			
Title	1	Identify the report as a systematic review.	Page 1
**ABSTRACT**			
Abstract	2	See the PRISMA 2020 for Abstracts checklist	
**INTRODUCTION**			
Rationale	3	Describe the rationale for the review in the context of existing knowledge.	Pages 2-3
Objectives	4	Provide an explicit statement of the objective(s) or question(s) the review addresses.	Page 3
**METHODS**			
Eligibility criteria	5	Specify the inclusion and exclusion criteria for the review and how studies were grouped for the syntheses.	Pages 3-5
Information sources	6	Specify all databases, registers, websites, organisations, reference lists and other sources searched or consulted to identify studies. Specify the date when each source was last searched or consulted.	Pages 3-4
Search strategy	7	Present the full search strategies for all databases, registers and websites, including any filters and limits used.	Page 3
Selection process	8	Specify the methods used to decide whether a study met the inclusion criteria of the review, including how many reviewers screened each record and each report retrieved, whether they worked independently, and if applicable, details of automation tools used in the process.	Page 4
Data collection process	9	Specify the methods used to collect data from reports, including how many reviewers collected data from each report, whether they worked independently, any processes for obtaining or confirming data from study investigators, and if applicable, details of automation tools used in the process.	Pages 4-5
Data items	10a	List and define all outcomes for which data were sought. Specify whether all results that were compatible with each outcome domain in each study were sought (e.g. for all measures, time points, analyses), and if not, the methods used to decide which results to collect.	Not applicable

(continued)

	10b	List and define all other variables for which data were sought (e.g. participant and intervention characteristics, funding sources). Describe any assumptions made about any missing or unclear information.	Pages 5-7, 11-16
Study risk of bias assessment	11	Specify the methods used to assess risk of bias in the included studies, including details of the tool(s) used, how many reviewers assessed each study and whether they worked independently, and if applicable, details of automation tools used in the process.	Pages 7, 16
Effect measures	12	Specify for each outcome the effect measure(s) (e.g. risk ratio, mean difference) used in the synthesis or presentation of results.	Not applicable
Synthesis methods	13a	Describe the processes used to decide which studies were eligible for each synthesis (e.g. tabulating the study intervention characteristics and comparing against the planned groups for each synthesis (item 5)).	Pages 3-5
	13b	Describe any methods required to prepare the data for presentation or synthesis, such as handling of missing summary statistics, or data conversions.	Not applicable
	13c	Describe any methods used to tabulate or visually display results of individual studies and syntheses.	Page 16
	13d	Describe any methods used to synthesize results and provide a rationale for the choice(s). If meta-analysis was performed, describe the model(s), method(s) to identify the presence and extent of statistical heterogeneity, and software package(s) used.	Not applicable
	13e	Describe any methods used to explore possible causes of heterogeneity among study results (e.g. subgroup analysis, meta-regression).	Not applicable
	13f	Describe any sensitivity analyses conducted to assess robustness of the synthesized results.	Not applicable
Reporting bias assessment	14	Describe any methods used to assess risk of bias due to missing results in a synthesis (arising from reporting biases).	Not applicable
Certainty assessment	15	Describe any methods used to assess certainty (or confidence) in the body of evidence for an outcome.	Not applicable
**RESULTS**			

(continued)

Study selection	16a	Describe the results of the search and selection process, from the number of records identified in the search to the number of studies included in the review, ideally using a flow diagram.	Pages 4, 10
	16b	Cite studies that might appear to meet the inclusion criteria, but which were excluded, and explain why they were excluded.	Not applicable
Study characteristics	17	Cite each included study and present its characteristics.	Pages 5-7, 11-15
Risk of bias in studies	18	Present assessments of risk of bias for each included study.	Pages 6, 15
Results of individual studies	19	For all outcomes, present, for each study: (a) summary statistics for each group (where appropriate) and (b) an effect estimate and its precision (e.g. confidence/credible interval), ideally using structured tables or plots.	Supplementary Tables S2, S3
Results of syntheses	20a	For each synthesis, briefly summarise the characteristics and risk of bias among contributing studies.	Pages 7, 16
	20b	Present results of all statistical syntheses conducted. If meta-analysis was done, present for each the summary estimate and its precision (e.g. confidence/credible interval) and measures of statistical heterogeneity. If comparing groups, describe the direction of the effect.	Not applicable
	20c	Present results of all investigations of possible causes of heterogeneity among study results.	Not applicable
	20d	Present results of all sensitivity analyses conducted to assess the robustness of the synthesized results.	Not applicable
Reporting biases	21	Present assessments of risk of bias due to missing results (arising from reporting biases) for each synthesis assessed.	Not applicable
Certainty of evidence	22	Present assessments of certainty (or confidence) in the body of evidence for each outcome assessed.	Pages 7, 16
**DISCUSSION**			
Discussion	23a	Provide a general interpretation of the results in the context of other evidence.	Pages 19-24
	23b	Discuss any limitations of the evidence included in the review.	Page 25
	23c	Discuss any limitations of the review processes used.	Not applicable

(continued)

	23d	Discuss implications of the results for practice, policy, and future research.	Page 25
**OTHER INFORMATION**			
Registration and protocol	24a	Provide registration information for the review, including register name and registration number, or state that the review was not registered.	Not applicable
	24b	Indicate where the review protocol can be accessed, or state that a protocol was not prepared.	Page 25
	24c	Describe and explain any amendments to information provided at registration or in the protocol.	Not applicable
Support	25	Describe sources of financial or non-financial support for the review, and the role of the funders or sponsors in the review.	Page 25
Competing interests	26	Declare any competing interests of review authors.	Not applicable
Availability of data, code and other materials	27	Report which of the following are publicly available and where they can be found: template data collection forms; data extracted from included studies; data used for all analyses; analytic code; any other materials used in the review.	Pages 2-3, Supplementary Tables S2, S3

PRIMSA Abstract Checklist

**TITLE**			
Title	1	Identify the report as a systematic review.	Yes
**BACKGROUND**			
Objectives	2	Provide an explicit statement of the main objective(s) or question(s) the review addresses.	Yes
**METHODS**			
Eligibility criteria	3	Specify the inclusion and exclusion criteria for the review.	No
Information sources	4	Specify the information sources (e.g. databases, registers) used to identify studies and the date when each was last searched.	Yes
Risk of bias	5	Specify the methods used to assess risk of bias in the included studies.	Yes
Synthesis of results	6	Specify the methods used to present and synthesize results.	Yes
**RESULTS**			
Included studies	7	Give the total number of included studies and participants and summarise relevant characteristics of studies.	Yes
Synthesis of results	8	Present results for main outcomes, preferably indicating the number of included studies and participants for each. If meta-analysis was done, report the summary estimate and confidence/credible interval. If comparing groups, indicate the direction of the effect (i.e. which group is favoured).	Yes
**DISCUSSION**			
Limitations of evidence	9	Provide a brief summary of the limitations of the evidence included in the review (e.g. study risk of bias, inconsistency and imprecision).	No
Interpretation	10	Provide a general interpretation of the results and important implications.	Yes
**OTHER**			
Funding	11	Specify the primary source of funding for the review.	Yes
Registration	12	Provide the register name and registration number.	No

From: Page MJ, McKenzie JE, Bossuyt PM, Boutron I, Hoffmann TC, Mulrow CD, et al. The PRISMA 2020 statement: an updated guideline for reporting systematic reviews. MetaArXiv. 2020, September 14. DOI: 10.31222/osf.io/v7gm2. For more information, visit: www.prisma-statement.org

Supplementary Table S2. Assessment of the quality of clinical trials GRADE.

№	Author, Year	TRP Channel	Design	Population (n)	IBD Subtype (UC/CD)	Method (IHC/WB/qPCR)	Sample Source/Cell Line	Clinical Outcomes	GRADE Level
1	Kun J. et al., 2014	TRPA1, TRPV1	Observational	12	UC and CD	IHC/qPCR	Samples from patients with active and inactive forms of CD and UC and healthy subjects	TRPA1 expression is significantly increased in patients with active IBD, but not inactive IBD, compared to non-inflamed samples; TRPV1 mRNA is significantly decreased in patients with active IBD compared to the non-inflamed group	Very low
2	Gombert S. et al., 2019	TRPA1	Observational	130	CD	Determination of CpG sites' methylation rates quantitative sensory testing (QST) pressure pain thresholds (PPT) patient-controlled analgesia (PCA)	Whole blood samples of CD patients and healthy participants	Increased TRPA1 promoter methylation correlates with dysregulated TRPA1 expression and enhanced peripheral pain sensitivity in CD patients	Low
3	Morita T. et al., 2020	TRPV1, TRPV2, TRPV3, TRPV4 TRPM2, TRPM4, TRPM5 TRPC1, TRPC3, TRPC4, TRPC5, TRPC6, TRPC7*	Observational	105	UC and CD	real-time PCR	Peripheral blood mononuclear cells (PBMCs) from patients with UC and CD and normal subjects	TRPV2 mRNA expression was negatively correlated with leukocyte count in UC; decreased TRPV2 mRNA expression levels in PBMCs of both UC and CD patients which negatively correlated with disease activity in both groups, suggesting a potential role in modulating inflammation	Very low
4	Luo C. et al., 2017	TRPV1	Observational	90	UC and CD	IHC	Colonic epithelium samples from patients with active forms of CD and UC and healthy controls	TRPV1 immunoreactivity was highly expressed on epithelial cells and infiltrating inflammatory cells in colon biopsies from patients with active IBD; however, TRPV1 expression did not correlate significantly with disease severity	Low
5	Akbar A. et al., 2010	TRPV1	Observational	68	UC and CD	Blood markers of inflammation (CRP, ESR) IHC/WB Short Form McGill Pain Questionnaire (SF-MPQ)	Samples from symptomatic and asymptomatic patients with quiescent IBD (CD, UC) and controls	TRPV1 expression correlated with abdominal pain severity	Low

						visual analogue scale (VAS)<br>Beck Depression Inventory (BDI)			
6	Toledo-Mauriño J. J. et al., 2018	TRPV1	Observational	53	UC	IHC/RT-PCR	Colonic tissue from patients with active and remission UC and non-IBD controls	Increased TRPV1 gene expression in remission UC patients compared to active UC patients; higher TRPV1 protein expression observed in all intestinal layers of active UC patients compared to non-IBD controls; upregulation of TRPV1 gene expression associated with earlier age at diagnosis and relapsing disease course	Low
7	Fichna J. et al., 2012	TRPV4	Observational	43	UC and CD	RT-PCR	Human colon biopsies from patients with CD and UC and healthy controls	TRPV4 mRNA expression was significantly elevated in patients with CD and UC compared with healthy subjects (2.9 and 4.5-fold, respectively)	Very low
8	Rizopoulos T., 2018	TRPV1, TRPV2, TRPV3, TRPV4	Observational	64	UC	IHC	Colonic tissue samples from patients with active and quiescent UC and non-IBD controls	No significant difference for TRPV3 expression levels between UC and control samples; TRPV4 expression levels were significantly increased in the colonic epithelium of UC patients compared to non-IBD controls; Statistically decreased TRPV1 expression levels were demonstrated for patients with active UC compared to the control group	Very low
9	Toledo Mauriño J. J. et al., 2020	TRPV2, TRPV3, TRPV4, TRPV5, TRPV6	Observational	71 (RT-PCR)<br>10 (IHC)	UC	RT-PCR<br>IHC	Colonic tissue from patients with active and remission UC and non-IBD controls	TRPV2-6 showed differential expression in UC patients compared to controls	Very low
10	Duo L. et al., 2020	TRPV1	Observational	90	UC and CD	IHC	Biopsy samples from UC and CD patients and controls	TRPV1 is highly expressed in patients with IBD	Very low
11	D'Aldebert E. et al., 2011	TRPV4	Observational	15	UC and CD	RT-PCR<br>IHC	Colonic tissue from patients with active CD, UC and non-IBD controls	TRPV4 is highly expressed in patients with IBD	Very low

TRP: Transient receptor potential channel; **IBD:** Inflammatory Bowel Disease; **CD:** Crohn's disease; **UC:** Ulcerative colitis; **PBMCs:** Peripheral blood mononuclear cells; **CRP:** C-reactive protein; **IHC:** immunohistochemistry; **WB:** western blot; **RT-PCR:** real time-polymerase chain reaction; **ESR:** erythrocyte sedimentation rate

Quality of evidence (GRADE Level): High-quality evidence suggests that it is unlikely that further research will change the confidence in the estimated effect; moderate-quality evidence suggests that further research is likely to have an important impact on confidence of the estimate and may change it; low-quality evidence means that further research is very likely to have an important impact on confidence in the estimation and may alter it; very low-quality evidence makes any estimate of the effect highly uncertain.

Supplementary Table S3. Assessment of the quality of preclinical studies (animal models) SYRCLE's Risk of Bias Tool and SPF status.

№	Author, Year	TRP Channel	Model	Intervention	Outcomes	SPF Status	SYRCLE Score
1	Kun J. et al., 2014	TRPA1, TRPV1	DSS-induced colitis WT and TRPA1 KO male mice	DSS	Protective role in colitis (TRPA1); the mechanisms underlying the anti-inflammatory action of TRPA1 in colitis may be explained by a decrease in the expression of proinflammatory receptors SP, NKA, NKB and NK1, as well as inhibition of the synthesis of inflammatory cytokines and chemokines, presumably originating from macrophages	🟡	Low
2	Kistner K. et al., 2016	TRPA1	DSS-induced colitis TRPV1 −/−	capsazepine (CPZ) or AITC enemas	Capsazepine (CPZ) and mustard oil (MO) activate TRPA1 channels in sensory neurons, resulting in persistent desensitization; This desensitization reduces the release of proinflammatory neuropeptides such as substance P and CGRP, thereby attenuating neurogenic inflammation	🔴	Unclear
3	Kurahara L. H. et al, 2018	TRPA1	TNBS-associated colitis TRPA1 KO mice	prednisolone treatment	In TRPA1 knockout mice, the extent of inflammation and fibrosis is more pronounced compared to wild-type mice	🔴	Unclear
4	Fichna J. et al., 2012	TRPV4	TNBS-associated colitis	TRPV4 antagonists: RN 1734, Ruthenium red mustard oil administration	Intestinal inflammation and colitis-associated pain; antagonists were used	🔴	Unclear
5	D'Aldebert E. et al., 2011	TRPV4	DSS-induced colitis ? 4αPDD-induced colitis	4α-PDD (4α-Phorbol 12,13-didecanoate) selective agonist of the TRPV4 ion channel	TRPV4 mRNA expression was up-regulated when compared with control naïve tissues	🔴	Unclear
6	Vermeulen W. et al., 2013	TRPA1, TRPV1	TNBS-associated colitis	BCTC (TRPV1's antagonist) TCS-5861528 (TRPA1's antagonist)	Visceral hypersensitivity; intraperitoneal blockade of TRPV1 plus TRPA1 further reduced the enhanced visceromotor responses to high distension pressures	🔴	Unclear
7	Mitrovic M. et al., 2010	TRPA1	DSS-induced colitis	intracolonic administration of AITC, capsaicin (5%) or their vehicle (peanut oil) TRPA1 channel blocker (HC-030031)	Visceral hypersensitivity mediated by TRPA1 agonist AITC	🟡	Unclear
8	Kimball E. S. et al., 2007	TRPA1	OM (Oil of mustard) colitis C57Bl/6 RAG2−/− mice	? OM application	Increased mRNA levels of various neuropeptides and mediators associated with pain and inflammation	🔴	Unclear
9	Brierley S. M. et al., 2009	TRPA1	TNBS-associated colitis Nodose and dorsal root (T10-L1, L6-S1) ganglia (wild-type and TRPA1−/− mice)	TRPA1 agonists (allylisothiocyanate-AITC; trans-cinnamaldehyde-TCA) Bradykinin capsaicin	TRPA1 mediates mechanosensitivy and pain hypersensitivity in colitis; its activation enhances responses to inflammatory stimuli; TRPA1 deficiency reduces colitis-associated pain	🔴	Unclear
10	Cattaruzza F. et al., 2010	TRPA1	TNBS-associated colitis wild-type and TRPA1−/− mice	TRPA1 agonists: mustard oil (MO), HNE sensitizing agents: PAR2 activating peptide (PAR2-AP)	TRPA1 expressed in colonic sensory neurons contributes to mechanosensitivity and visceral pain hypersensitivity in colitis; TRPA1 deficiency attenuates colitis-associated pain responses	🟡	Unclear

11	Jain P. et al., 2020	TRPA1	DSS-induced colitis<br>Trpa1 −/− mice	AITC (TRPA1 agonist)<br>HC-030031 (TRPA1 antagonist)	TRPA1 contributes to colitis-associated mechanical hypersensitivity in somatic; tissues TRPA1 deficiency or pharmacological blockade reduces this hypersensitivity without affecting colitis severity; DSS treatment increases TRPA1 expression and responsiveness in DRG nociceptors	🟡	Low
12	Kumar V. et al., 2022	TRPA1, TRPV1	C57BL/6 mice treated with intrarectal capsazepine	CPZ<br>SCFA (short chain fatty acids)	Intra rectal administration of capsazepine modulates TRPA1/TRPV1-positive nociceptors (behavioral pain assays) and resulted in damaged mucosal lining, increased gut permeability, and altered transcriptional profile of genes for goblet cell markers, mucus regulation, immune response, and tight junction proteins	🟡	Unclear
13	Li Q. et al., 2016	TRPA1	TNBS-associated colitis	TNBS	TRPA1 expression is upregulated in the spinal dorsal horn following TNBS-induced colitis, correlating with increased visceral hypersensitivity	🟡	Low
14	Yang Y. et al., 2019	TRPA1	DSS-induced colitis<br>Trpa1 -KO	TRPA1 agonists: allyl isothiocyanate (AITC) and cinnamaldehyde<br>TRPV1 agonist: capsaicin<br>TRPA1 selective antagonists: A967079 and HC030031	In DSS-induced colitis, TRPA1 expression is upregulated, and TRPA1 activation exacerbates abnormal colonic motility; pharmacological or genetic inhibition of TRPA1 alleviates these motility disturbances	🟡	Unclear
15	Nishiyama K. et al., 2024	TRPC1-7	DSS-induced colitis<br>TRPC3 KO<br>TRPC6 KO	PPZ2 treatment	TRPC6 expression increased in DSS-induced colitis; TRPC6 KO mice exhibited severe weight loss, increased disease activity index (DAI), and reduced Zn^{2+} levels compared to wild-type (WT) mice; treatment with TRPC6 activator PPZ2 prevented DSS-induced colitis progression, increased Zn^{2+} concentration, and suppressed IL-6 expression in the colon; TRPC6-mediated Zn^{2+} influx plays a key role in stress resistance against IBD	🟡	Unclear
16	Matsumoto K. et al., 2016	TRPM2	TNBS-associated colitis<br>Sprague-Dawley rats<br>TRPM2−/− mice	TRPM2 inhibitor: econazole	Increased visceromotor reflexes caused by balloon pressure, visceral hypersensitivity	🟡	Unclear
17	Yamamoto S. et al., 2008	TRPM2	Trpm2-KO mice monocytes<br>DSS-induced colitis	DSS	Progression of colitis through its possible implication in oxidative stress signaling	🔴	Unclear
18	Nakamoto T. et al., 2024	TRPM2	Trpm2-KO mice monocytes<br>TNBS-associated colitis<br>BMDM	H2O2	TRPM2 contributes to inflammation via Th1/Th17 pathways; TRPM2 mediates ROS-induced cytokine release and MAPK activation	🟡	Unclear
19	King J. W. et al., 2024	TRPM3	DSS-induced colitis	TRPM3 agonists CIM-0216 capsaicin, and high K+<br>TRPM3 inhibitor isosakuranetin	Perception of noxious stimuli in colitis, colonic hypersensitivity	🟡	Unclear
20	Hosoya T. et al., 2014	TRPM8	DSS-induced colitis<br>TNBS-associated colitis	TRPM8 agonist, WS-12<br>TRPM8 channel blocker AMTB	Visceral hyperalgesia	🟡	Low
21	Ramachandran R.	TRPM8	DSS-induced colitis	icilin	Anti-inflammatory role of TRPM8 activation, partly mediated	🟡	Unclear

	et al., 2013		TRPM8 KO mice		by inhibition of neuropeptide release		
22	de Jong P. R. et al., 2015	TRPM8	DSS-induced colitis<br>TNBS-associated colitis(доп.)<br>Trpm8−/− mice<br>Trpm8−/− CD11c+ DCs (dendritic cells)<br>Nk1 −/−, Ramp1 −/−<br>Trpm8 −/− BMDC	(LPS; TLR4 ligand) or CpG-ODN (TLR9 ligand) recombinant CGRP (rCGRP) pretreatment	TRPM8 expressed on mucosal sensory neurons regulates colitogenic responses by innate immune cells via CGRP; TRPM8 deficiency leads to increased susceptibility to colitis, while CGRP administration ameliorates inflammation	🔴	Unclear
23	Khalil M. et al., 2016	TRPM8	Trpm8 tm1Jul /J<br>DSS-induced colitis<br>murine PM and BMDM<br>TRPM8-deficient macrophage transfer	LPS<br>menthol (TRPM8 agonist)	TRPM8 expression in macrophages regulates the balance between pro-inflammatory TNF-α and anti-inflammatory IL-10 production; TRPM8 deficiency leads to increased colitis severity, while activation of TRPM8 with menthol enemas provides protection; adoptive transfer of TRPM8-deficient macrophages exacerbates colitis, whereas systemic IL-10 overexpression mitigates this effect	🔴	Unclear
24	Zhang Z. et al., 2024	TRPM8	DSS-induced colitis<br>ND7/23	menthol (TRPM8 agonist)<br>Aprepitant	TRPM8 activation by menthol induces calcium influx in DRG neurons and inhibits substance P (SP) release via suppression of PKA and GSK-3β pathways. This modulation reduces SP-mediated epithelial apoptosis in colonic organoids and alleviates DSS-induced colitis symptoms in mice. Combined treatment with menthol and the SP receptor antagonist Aprepitant shows no additive effect, suggesting a shared therapeutic mechanism	🔴	Unclear
25	Matsumoto K. et al., 2023	TRPV2 and TRPV1	TNBS-induced colitis	TRPV1 antagonists: capsazepin, BCTC, SB-366791, AMG9810, and JNJ-17203212<br>TRPV2 antagonists: tranilast, SKF96365	Visceral hypersensitivity	🟡	Unclear
26	Duo L. et al., 2020	TRPV1	DSS-induced colitis<br>Trpv1G564S+/+ mice	LPS, OVA 323–339, TGF-β and IL-2<br>calcineurin inhibitor FK506	TRPV1 gain of function significantly increased the susceptibility of<br>mice to experimental colitis, that was associated with excessive recruitment of dendritic cells and enhanced Th17 immune responses in the lamina propria of colon; TRPV1 gain of function promoted dendritic cell activation and cytokine production upon inflammatory stimuli, and consequently enhanced<br>dendritic cell-mediated Th17 cell differentiation	🔴	Low
27	Lapointe T. K. et al., 2015	TRPV1	DSS-induced colitis<br>TRPV1−/− mice<br>Transgenic Ai32/TRPV1-cre mice	HEK 293 tsA201 (SP, broad-spectrum PKC inhibitor GF109203X, PKC agonist phorbol myristate acetate); capsaicin	Increased inflammation, increased release of CGRP and SP, visceral hypersensitivity and pain-related behavior	🟡	Unclear
28	Engel M. A. et al., 2012	TRPV1	DSS colitis<br>CGRP−/−, SP−/−<br>RTX-desensitized WT mice	capsaicin	Increased inflammation, increased release of CGRP and SP	🔴	Unclear

29	Utsumi D. et al., 2018	TRPA1, TRPV1 (diff)	DSS colitis<br>TRPV1KO<br>TRPA1KO<br>BM-chimeric mice	capsaicin	TRPV1 and TRPA1 expression in sensory neurons plays a critical role in the progression of colonic inflammation in DSS-induced colitis in mice	🟡	Unclear
30	Lee J. et al., 2012	TRPV1	oxazolone-induced colitis	capsaicin	Excessive neutrophil accumulation; a protective role of TRPV1 expressing extrinsic sensory neurons in oxazolone induced colitis	🟡	Unclear
31	Massa F. et al., 2006	TRPV1	DNBS-induced colitis<br>TRPV1−/− mice	DNBS, atropine	Modulating of sensory pathways involved in colonic inflammation, possible protective effect	🟡	Unclear
32	Chen J., 2013	TRPA1	DNBS-induced colitis	HeICS	Chronic stress, following colitis, upregulates TRPA1 and NGF in colonic tissues, downregulates Kv1.1 and Kv1.4 in DRG neurons, leading to increased visceral hypersensitivity; TRPA1 antagonism mitigates these effects	🔴	Unclear
33	De Schepper H. U. et al., 2008	TRPV1	TNBS	TRPV1 antagonists: capsazepine, and N-(4-tertiarybutylphenyl)-4-(3-cholorphyridin-2-yl)tetrahydropyrazine-1(2H)carboxamide (BCTC)<br>CGRP antagonist: CGRP-(8-37);<br>capsaicin	TRPV1 receptor activation mediates afferent nerve sensitization during colitis-induced motility disorders in rats. Inhibition of TRPV1 signaling reduces colitis-induced motility disorders and afferent nerve sensitization	🔴	Unclear
34	De Schepper H. U. et al., 2008 (2)	TRPV1	TNBS	BCTC	TRPV1 receptors on unmyelinated C-fibres mediate colitis-induced sensitization of pelvic afferent nerve fibres in rats. Inhibition of TRPV1 signaling reduces colitis-induced sensitization and associated pain	🔴	Unclear
35	Matsumoto K. et al., 2012	TRPV1	DSS-induced colitis	BCTC<br>5-HT3 antagonist: alosetron	DSS-induced colitis leads to increased TRPV1 and 5-HT_3 receptor expression and decreased 5-HT_4 receptor expression in colonic mucosa, contributing to visceral hypersensitivity	🟡	Unclear
36	Mazor Y. et al., 2024	TRPV1	DNBS-induced colitis	QX-314<br>capsaicin;<br>capsazepine	Intrarectal co-application of QX-314 (a membrane-impermeable sodium channel blocker) with capsaicin selectively silences TRPV1-expressing nociceptors, significantly reducing colitis-induced visceral hypersensitivity and ongoing pain. Notably, QX-314 alone was effective in inflamed tissue, indicating tonic TRPV1 activity in colitis. These effects were absent when TRPV1 channels were blocked, underscoring their critical role in mediating colitis-associated pain	🟡	Unclear
37	Miranda A. et al., 2007	TRPV1	TNBS	capsaicin;<br>JYL1421	TNBS-induced colitis upregulates TRPV1 expression in dorsal root ganglia, leading to increased visceral sensitivity to mechanical and chemical stimuli. Preemptive administration of the TRPV1 antagonist JYL1421 reduces both inflammation and hypersensitivity; post-inflammatory treatment attenuates mechanical, but not chemical, hypersensitivity	🟡	Unclear

38	Shen S. et al., 2017	TRPV1	TNBS	RTX<br>PI3K LY294002	Nerve growth factor (NGF) upregulates TRPV1 protein expression in DRG neurons via the PI3K/Akt signaling pathway. Inhibition of PI3K/Akt signaling reduces TRPV1 expression and alleviates colitis-induced visceral hypersensitivity	🟡	Unclear
39	Wu Y. et al., 2019	TRPV1	TLR4 KO<br>2,4,6-trinitrobenzene sulfate-induced colit	Capsaicin-evoked TRPV1 currents	TLR4 deficiency in TNBS-induced colitis mice resulted in reduced TRPV1 gene and protein expression in DRG neurons, decreased capsaicin-induced TRPV1 current density, and alleviated visceral hypersensitivity compared to WT mice. TLR4 signaling contributes to TRPV1 upregulation and peripheral sensitization in inflammatory conditions	🟡	Unclear
40	Issa C. M. et al., 2014	TRPV2	DSS<br>TRPV2−/− mice	DSS	Increased inflammation	🟡	Unclear
41	Matsumoto K. et al., 2018	TRPV4	DSS-induced colitis<br>TRPV4 KO	GSK1016790A (agonist)	Pro-inflammatory effects	🟡	Low

TRP: Transient receptor potential channel; **DSS:** Dextran Sulfate Sodium; **DNBS:** Dinitrobenzene sulfonic acid; **TNBS:** 2,4,6-Trinitrobenzene sulfonic acid; **OM:** Oil of mustard; **AITC:** Allyl isothiocyanate; **DRG:** Dorsal root ganglia neurons; **CGRP**: Calcitonin Gene-Related Peptide; **SP:** Substance P; **5-HT_3 receptor:** $5\text{-hydroxytryptamine}_3$ receptor; **5-HT_4 receptor:** $5\text{-hydroxytryptamine}_4$ receptor; **MAPK:** Mitogen-activated protein kinase expression and decreased; **Th1/Th17:** T helper 1/T helper 17; **ROS:** Reactive oxygen species; **TLR4:** Toll-like receptor 4; **LPS:** lipopolysaccharide

Risk of Bias (SYRCLE Score): Low risk of bias was assigned when the methodological approach for a given domain was explicitly described and met the SYRCLE's RoB criteria; high risk of bias was assigned when the study clearly indicated that the methodological safeguard was not implemented; unclear risk of bias was assigned when there was insufficient information to determine whether the safeguard was implemented.

SPF Status: Red circle 🔴 – studies with unrestricted animal care or without confirmed SPF status, classified as high risk of bias; yellow circle 🟡 – studies with confirmed SPF status but insufficient information on animal care, classified as moderate risk of bias; green circle 🟢 – studies meeting all predefined SPF and animal care criteria, classified as low risk of bias.
